# From Detoxified Yam to Bioactive Extracts: Integrated Extraction and In Silico Evidence of Anti-Biofilm Activity of *Dioscorea hispida* Extracts Against *Cutibacterium acnes*

**DOI:** 10.3390/life16091494

**Published:** 2026-09-06

**Authors:** Suthinee Sangkanu, Jiraporn Khanansuk, Muhammad Ikhlas Abdjan, Yan Wang, Sathianpong Phoopha, Wandee Udomuksorn, Michael Wink, Sukanya Dej-adisai

**Affiliations:** 1 Department of Pharmacognosy and Pharmaceutical Botany, Faculty of Pharmaceutical Sciences, Prince of Songkla University, Hat Yai 90112, Songkhla, Thailand; suthinee.s@psu.ac.th (S.S.); jiraporn.kha@psu.ac.th (J.K.); 2 Department of Chemistry, Faculty of Mathematics and Natural Science, Universitas Negeri Surabaya, Surabaya 60231, Indonesia; muhammadabdjan@unesa.ac.id; 3 Key Laboratory for Chemistry and Molecular Engineering of Medicinal Resources (Ministry of Education of China), Guangxi Key Laboratory of Chemistry and Molecular Engineering of Medicinal Resources, University Engineering Research Center for Chemistry of Characteristic Medicinal Resources (Guangxi), School of Chemistry and Pharmaceutical Sciences, Guangxi Normal University, Guilin 541004, China; wy198565@gxnu.edu.cn; 4 Traditional Thai Medical Research and Innovation Center, Faculty of Traditional Thai Medicine, Prince of Songkla University, Hat Yai 90112, Songkhla, Thailand; sathianpong.p@psu.ac.th; 5 Division of Health and Applied Science, Faculty of Science, Prince of Songkla University, Hat-Yai 90112, Songkhla, Thailand; wandee.u@psu.ac.th; 6 Institute of Pharmacy and Molecular Biotechnology, Heidelberg University, 69120 Heidelberg, Germany; wink@uni-heidelberg.de

**Keywords:** *Dioscorea hispida*, biofilm, *Cutibacterium acnes*, _CA_Lipase, docking, metabolites

## Abstract

The increasing prevalence of biofilm-associated infections caused by *Cutibacterium acnes* has stimulated interest in food-derived natural products as alternative sources of anti-biofilm agents. This study investigated the effects of processing and extraction conditions on the phytochemical composition, antibacterial activity, and anti-biofilm properties of *Dioscorea hispida* Dennst. Reflux extraction of dried yam with 80% ethanol produced the crude extracts with the highest yields (1.39–1.80%), whereas fresh yam yielded 0.51–0.97% extract. Using Gas–liquid chromatography–mass spectrometry (GLC-MS) analysis, linoleic acid ethyl ester, n-hexadecanoic acid, 9,12-octadecadienoic acid (Z,Z)-, and stigmasterol were identified as the major constituents. Among the tested extracts, DH-W-F-H (*D. hispida*-water washing-fresh-hexane) and DH-W-F-E (*D. hispida*-water washing-fresh-ethanol) were extracted from fresh yam using hexane and ethanol, respectively, while DH-W-D-E (*D. hispida*-water washing-dry-ethanol) was isolated from dried yam using ethanol and exhibited the strongest antibacterial activity, with minimum inhibitory concentrations (MIC) ranging from 64 to 2048 µg/mL. These extracts demonstrated pronounced concentration-dependent inhibition of biofilm formation by *Staphylococcus epidermidis*, *Staphylococcus aureus*, and *Cutibacterium acnes*. The strongest anti-biofilm activity was observed against *C. acnes*, with biofilm formation nearly eliminated at MIC concentrations. Moreover, all three extracts significantly reduced established *C. acnes* biofilms, with DH-W-F-H exhibiting greater eradication efficacy than vancomycin under the tested conditions. To elucidate the underlying mechanism, major fatty acid derivatives were evaluated against *C. acnes* lipase (_CA_Lipase), a virulence factor associated with biofilm development, using molecular docking, molecular dynamics simulations, and the Molecular Mechanics-Generalized Born Surface Area (MM-GBSA) binding free-energy calculations. The compounds exhibited favorable interactions with _CA_Lipase, with linoleic acid ethyl ester (FA2) showing the strongest binding affinity, stable protein–ligand interactions throughout a 200 ns simulation, and the most favorable binding free energy. Collectively, the biological and computational findings suggest that fatty acid-rich extracts from processed *D. hispida* suppress biofilm formation through an antivirulence mechanism involving _CA_Lipase inhibition. These results highlight the potential of *D. hispida* as a source of metabolites for the development of functional food ingredients and value-added cosmetic and dermatological applications.

## 1. Introduction

*Cutibacterium acnes* (formerly known as *Propionibacterium acnes*) is an anaerobic Gram-positive bacterium that is the most prevalent bacterial strain inhabiting human skin, especially in sebaceous areas characterized by high sebum production [1]. Several virulence mechanisms appear to be mediated by *C. acnes*, including the secretion of hydrolytic enzymes such as lipases and hyaluronidases, which promote follicular disruption and inflammation [2]. Inflammation and the onset of acne vulgaris are caused by the overgrowth of *C. acnes* in closed hair follicles. Acne vulgaris is the most common skin condition among adolescents and a widespread dermatological issue. It affects roughly 50 million people in the United States [3]. Furthermore, the ability of *C. acnes* to form biofilms is recognized as a key virulence trait that enhances its antibiotic resistance [4]. The biofilm-forming capacity of *C. acnes* was first demonstrated in vitro in 2007 [5], and subsequent studies have revealed that biofilm development is temperature-dependent, reaching its optimum at 37 °C [6]. Subsequent investigations showed that phylotypes IA and II can form extensive biofilm-associated macrocolonies within sebaceous follicles [7]. Accumulating evidence indicates that biofilm formation is a critical virulence determinant of *C. acnes* and plays a central role in acne pathogenesis. Furthermore, biofilm production varies among phylogenetic groups, with phylotype IA1 exhibiting the highest biofilm-forming capacity, followed by phylotypes IC, IA2, and II, whereas phylotypes IB and III generally show lower biofilm development [8]. The extracellular polymeric matrix produced during biofilm formation contains exopolysaccharides that act as a biological adhesive, promoting corneocyte aggregation and increasing resistance to conventional antibiotic therapies, thereby contributing to the progression of chronic inflammation [9]. For example, *C. acnes* has additionally been found in cases of breast implant infections linked to biofilm formation that triggers chronic inflammation [10], as well as prosthetic valve endocarditis from cardiovascular devices [11]. Consequently, the exploration of novel bioactive compounds targeting *C. acnes* and its virulence mechanisms, especially biofilm formation, has emerged as a promising research direction. In cosmetic dermatology, anti-biofilm strategies offer significant potential to prevent and manage acne by suppressing bacterial pathogenicity rather than relying solely on conventional antibiotic therapies. This approach may help reduce antibiotic consumption and mitigate the growing challenge of antimicrobial resistance.

*Dioscorea* species, commonly known as yams, are monocotyledonous, tuberous crops in the family Dioscoreaceae, comprising over 630 distinct species worldwide [12]. *D. hispida* is native to Southeast Asia and north-east Australia. It is cultivated primarily in the tropical and subtropical regions of Asia, Africa, the Americas, and Australia. In the Philippines, *D. hispida* is known as namò and kuyot in the Luzon and Visayan regions, respectively; in Malaysia, it is known as umbi gadung; and in Thailand, it is known as kloi and is commonly known as intoxicating, or wild yam. Despite its toxicity, due to a high oxalate and dioscorine content in the raw state, yam has long been utilized as a traditional food resource in several Southeast Asian countries following appropriate detoxification processes. Traditional methods such as soaking, washing, fermentation, and prolonged exposure to running water are used to reduce toxic compounds to safer levels. Such traditional utilization highlights the potential of yam as an underexploited food crop with added value beyond its nutritional role [13].

In terms of nutritional benefits, *D. hispida* is considered a plant with complete nutrients, including carbohydrates, protein, fiber, and various vitamins and minerals [14]. In addition to their nutritional value, numerous *Dioscorea* species possess considerable medicinal and economic potential. The bioactivity of *D. hispida* extracts is primarily attributed to their high levels of phenolic and flavonoid compounds. These secondary metabolites exhibit significant antioxidant and anti-inflammatory potential [15]. *D. hispida* produces a wide range of bioactive secondary metabolites that may contribute to its pharmacological characteristics, in addition to fatty acid, phenolic and flavonoid components. Alkaloids, steroidal saponins, steroidal sapogenins (including derivatives of diosgenin), terpenoids, phytosterols, tannins, and steroidal substances have all been found in various plant parts by phytochemical analyses. Among the most pharmacologically significant components of the genus *Dioscorea* are steroidal saponins and their aglycones, which have anti-inflammatory, hypolipidemic, antibacterial, and anticancer properties [16]. Alkaloids, such as the toxic alkaloid dioscorine and the cyanogenic glucosides linamarin and lotaustralin found in *D. hispida*, support the biological activity of the plant but also cause its well-known toxicity prior to processing [17]. Terpenoids, phytosterols, and tannins further enhance the antioxidant, antimicrobial, and anti-inflammatory potential of the species, highlighting *D. hispida* as a promising source of multifunctional phytochemicals for pharmaceutical and nutraceutical applications [18]. Fatty acids are particularly relevant to antimicrobial activity because their antibacterial effects are influenced by structural characteristics such as carbon-chain length and degree of unsaturation. They can interact with microbial membranes and interfere with membrane integrity and cellular functions, thereby contributing to microbial inhibition [19].

Several species within the genus *Dioscorea* have been reported to exhibit notable antimicrobial activity. For instance, extracts of *D. bulbifera*, *D. hispida*, *D. glabra*, and *D. pentaphylla* have demonstrated inhibitory effects against a range of pathogenic microorganisms, including *Escherichia coli*, *Streptococcus pneumoniae*, *Klebsiella pneumoniae*, *Shigella dysenteriae*, *Candida albicans*, and *Candida tropicalis* [20]. In addition, the findings demonstrate that *D. bulbifera* exhibited considerable potential as a source of anti-biofilm agents. Through a green synthesis approach, extracts of *D. bulbifera* were successfully utilized to produce gold–silver core–shell nanoparticles that exhibited strong inhibitory effects against biofilm-forming Gram-negative and Gram-positive bacteria, particularly *Acinetobacter baumannii* and *S. aureus* [21]. While antimicrobial properties have been documented in various *Dioscorea* species, studies evaluating the activity of *D. hispida* against *C. acnes* remain limited. In addition, the inhibitory effects of *D. hispida* on *C. acnes* biofilm development, as well as the phytochemical compounds underlying these activities, have not been extensively investigated. Therefore, the present study aimed to characterize the volatile phytochemical constituents of *D. hispida* extracts and evaluate their antibacterial and anti-biofilm activities against *C. acnes*. The findings may provide scientific evidence supporting the use of this underexplored yam species as a source of bioactive compounds for cosmetic and health-related applications.

## 2. Materials and Methods

### 2.1. Chemical and Media

Dimethyl sulfoxide and hexane were obtained from RCI Labscan Limited (Bangkok, Thailand), while ethanol was purchased from Thaioil Co., Ltd. (Bangkok, Thailand). Mueller–Hinton agar (MHA), Mueller–Hinton broth (MHB), and Brain Heart Infusion medium (BHI) were obtained from Merck (Darmstadt, Germany). D-Glucose was purchased from HiMedia Laboratories (Mumbai, India), and crystal violet was obtained from PanReac AppliChem (Barcelona, Spain). Resazurin and sodium chloride were purchased from Sigma-Aldrich (St. Louis, MO, USA) and Kemaus (Cherrybrook, NSW, Australia), respectively.

### 2.2. Preparation of D. hispida Extracts

**Figure 1:**
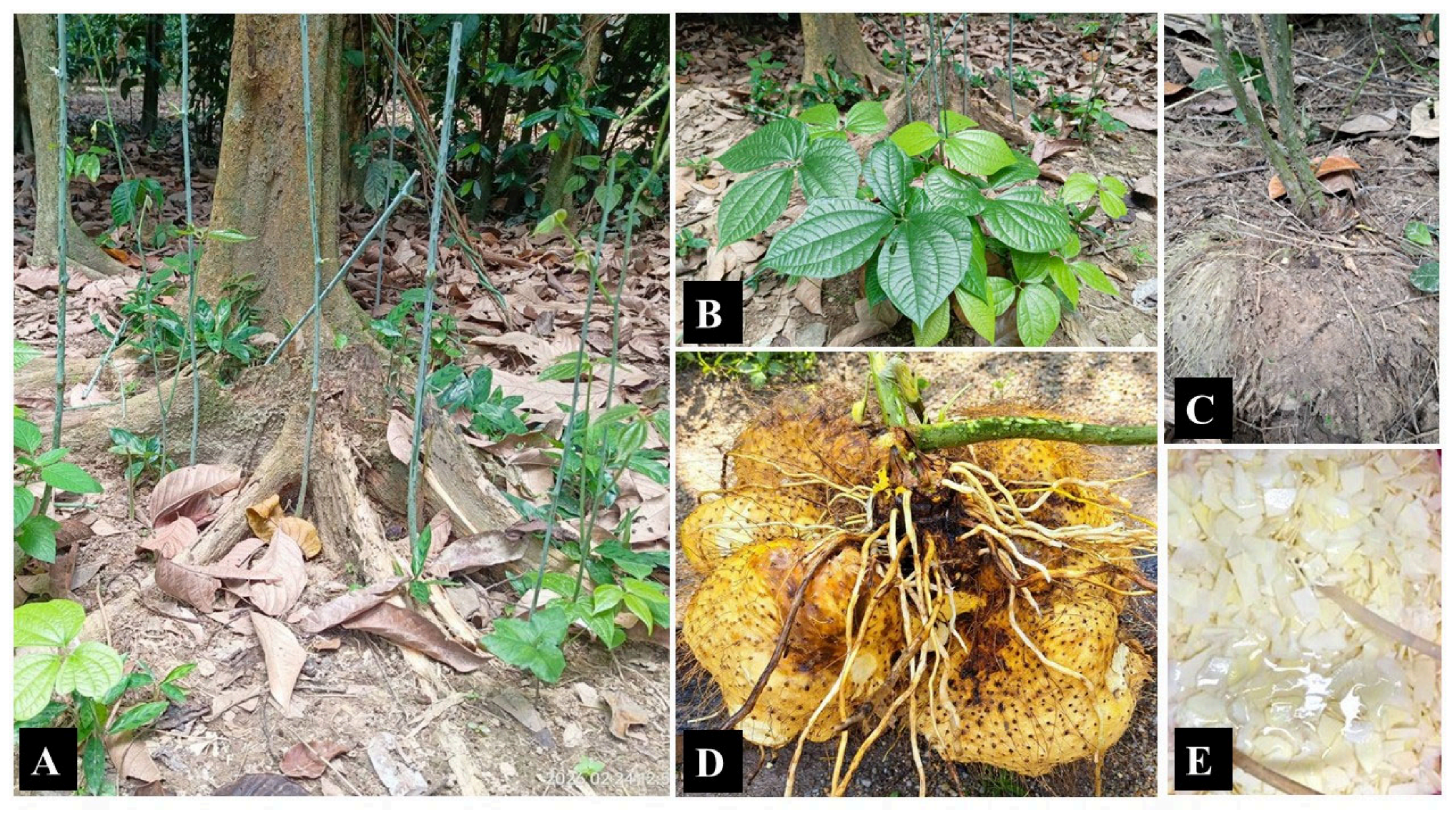
Wild yam (*D. hispida* Dennst.) Stems (**A**). Leaves with three veins per leaflet (**B**). Tuber located within the soil in the forest (**C**). Wild yam tubers are characterized by a brown outer peel and are covered with rigid roots (**D**). Small fragments of tuber submerged in water during washing to remove dioscorine (**E**).

Tubers of *Dioscorea hispida* Dennst., commonly known as yam, were collected from Nakhon Si Thammarat Province, southern Thailand (8.3389° N, 99.8511° E) (Figure 1). The plant specimen was taxonomically identified, and a voucher specimen was deposited in the herbarium of the Department of Pharmacognosy and Pharmaceutical Botany, Faculty of Pharmaceutical Sciences, Prince of Songkla University, under herbarium number SKP 062 04 08 001. The tubers were thoroughly washed with tap water to remove adhering soil, debris, and other contaminants, then peeled and cut into small pieces. The prepared samples were subsequently divided into two experimental groups. For Group 1, a dioscorine-removal process was performed by immersing the yam pieces in saline solution for 24 h, then rinsing them under running water for an additional 24 h. This washing procedure was repeated three times. The effectiveness of dioscorine removal was assessed using seven alkaloid detection reagents, namely Dragendorff’s, Marquis, Mayer’s, Sonnenschein’s, Wagner’s, tannic acid, and Hager’s reagents. Group 2 samples were processed without any detoxification treatment and served as untreated yam samples.

**Figure 2:**
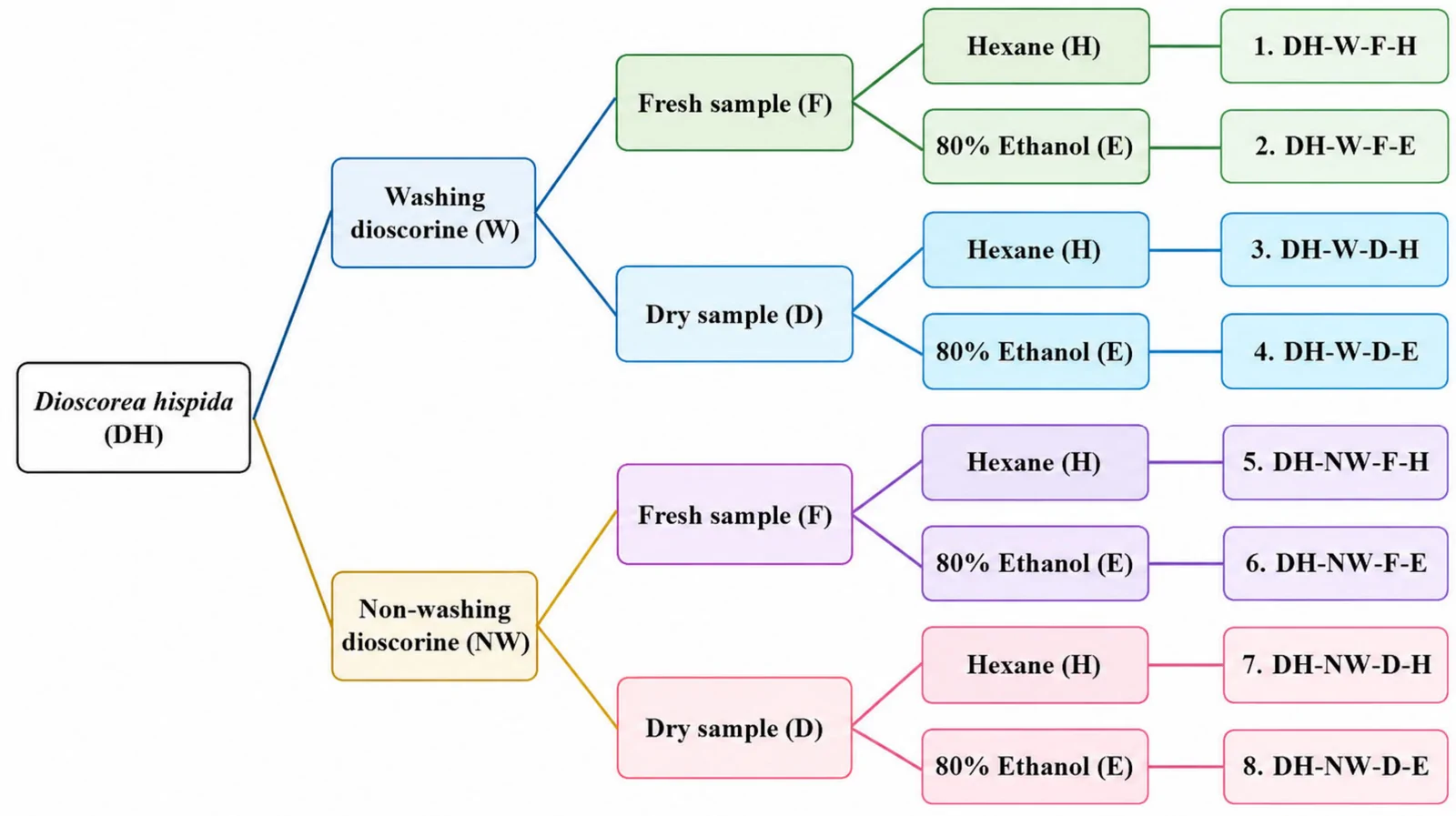
Scheme for *D. hispida* preparation.

Before extraction, samples from both groups were further divided into fresh and dried samples (Figure 2). The dried samples were maintained at 50 °C for 3 days to reduce moisture while minimizing potential degradation of heat-sensitive phytochemicals. The comparison between fresh and dried materials was performed to determine whether sample moisture status and the drying process influence the recovery and biological activity of *D. hispida* constituents. Extraction was performed by reflux for 5 h using either 80% ethanol or hexane to obtain extracts with different chemical profiles based on solvent polarity. The 80% ethanol was selected to extract predominantly polar and moderately polar constituents, such as phenolic and flavonoid compounds, whereas hexane was used to recover relatively nonpolar and lipophilic constituents. The reflux method was selected to enhance solvent penetration and solute transfer under controlled heating, thereby facilitating efficient extraction. Following extraction, the solvent phase was collected, filtered through filter paper, and concentrated by solvent evaporation. The resulting crude extracts were transferred to airtight containers and stored at 4 °C until further analysis.

### 2.3. Gas–Liquid Chromatography–Mass Spectrometry Analysis

The eight *D. hispida* extracts were sent to the Office of Scientific Instrument and Testing (OSIT) at Prince of Songkla University for GLC-MS analysis [22]. The analysis was conducted using a 7820A gas chromatograph linked to a 5975C network mass spectrometer (GLC–MS) from Agilent Technologies (Waldbronn, Baden-Württemberg, Germany). The separation was performed on an Agilent Technologies HP-5MS column, which consists of 5% Phenyl Methyl Siloxane and has a 30 m × 250 µm × 0.25 µm dimension. The column temperature was initially set to 80 °C and held for three minutes, then increased to 280 °C at 5 °C/min and maintained at that temperature for five minutes. The entire run took 48 min. A split ratio of 10:1 was used, along with an injection volume of 1 µL. Helium served as the carrier gas at a flow rate of 1 mL/min. Mass spectrometry detection was performed using electron ionization (EI) at 70 eV and in full-scan acquisition mode, covering a mass-to-charge (*m*/*z*) range of 35–350. The authors only considered compounds that made up more than 1% of the total detected substances and had a matching factor of over 90% from the database.

### 2.4. Antibacterial Activity

#### 2.4.1. Bacterial Inoculum

Bacterial inocula were prepared by culturing *S. aureus* ATCC 25923 and *S. epidermidis* TISTR 517 on Mueller-Hinton Agar (MHA) at 37 °C for 24 h. A few colonies were transferred into Mueller–Hinton broth (MHB) and incubated at 37 °C for 3–5 h. The bacterial suspension was then standardized to a turbidity equivalent to a 0.5 McFarland standard and subsequently diluted 1:200 in MHB to obtain a final concentration of approximately 1 × 10^6^ CFU/mL. *C. acnes* DMST 14916 was cultured on Brain Heart Infusion Agar (BHIA) at 37 °C for 72 h under aerobic conditions. Then, the culture was further adjusted to a turbidity equivalent to the 0.5 McFarland standard and further diluted 1:200 in Brain Heart Infusion Broth (BHIB) to achieve a target concentration of approximately 1 × 10^6^ CFU/mL.

#### 2.4.2. Antibacterial Screening

The extracts were prepared at a final concentration of 2 mg/mL and initially tested against various bacteria using a colorimetric broth microdilution method in 96-well microtiter plates, following guidelines from [23] with some modifications. The stock solutions of the extracts (200 mg/mL) were diluted to 4 mg/mL using MHB or BHIB. Subsequently, 50 µL of each diluted extract was added to each well in triplicate. To this, 50 µL of *S. aureus* ATCC 25923 and *S. epidermidis* TISTR 517 inocula (10^6^ CFU/mL) were added, resulting in a final extract concentration of 2 mg/mL. Plates containing all bacterial strains were incubated at 37 °C for 15 h, while *C. acnes* DMST 14916 was incubated in an anaerobic jar at 37 °C for 72 h. After incubation, 20 µL of a 0.1% resazurin solution was added to each well, and the plates were incubated for an additional 3 h, following a modified protocol from [24]. A negative result was identified by a change in resazurin color from purple to pink, whereas retention of a blue or purple color indicated inhibition of bacterial growth and was therefore considered a positive result. Vancomycin at a concentration of 30 µg/mL was used as a positive control.

#### 2.4.3. Determination of Minimum Inhibitory Concentration (MIC) and Minimum Bactericidal Concentration (MBC)

The CLSI guidelines [23,24] were adapted to evaluate the minimum inhibitory concentrations (MICs) and minimum bactericidal concentrations (MBCs) of extracts. Ten sample concentrations were prepared through two-fold serial dilutions, ranging from 2048 to 4 µg/mL, and each was tested in triplicate. The MIC was defined as the lowest concentration of extract that resulted in no color change (specifically blue) in the resazurin indicator. To determine the MBC, all wells that showed positive results were sub-cultured onto Mueller-Hinton agar (MHA) or Brain Heart Infusion agar (BHIA) plates and incubated under suitable conditions. The MBC was established as the lowest extract concentration that exhibited no visible bacterial growth. All experiments were conducted in triplicate.

### 2.5. Inhibition of Biofilm Formation

For the assessment of biofilm formation inhibition, *C. acnes* DMST 14916 was cultured for 72 h in Brain Heart Infusion Broth (BHIB) + 1% glucose (BHIB_glu_) under anaerobic conditions. *S. aureus* ATCC 25923 and *S. epidermidis* TISTR 517 were inoculated into MHB + 1% glucose (MHB_glu_) and incubated at 37 °C for 24 h. The bacterial culture was subsequently diluted with BHIB_glu_ and MHB_glu_ to an appropriate concentration (1 × 10^8^ CFU/mL), and 100 μL of this suspension was inoculated into the wells of a sterile 96-well flat-bottom microplate (Nunclon Surface F; Nunc A/S) [25]. Three concentrations of the test extracts were added to achieve a final volume of 200 μL/well and final concentrations of 0.25 × MIC, 0.5 × MIC, and MIC. Each concentration was tested in triplicate, with an additional well designated as a blank control for the extract. Controls included a positive biofilm formation control containing only bacterial strains, and a background control containing only medium. Then, the microplate was incubated for 72 h under anaerobic conditions and 24 h under aerobic conditions to facilitate biofilm development. Post-incubation, bacterial growth was quantified by measuring optical density at 600 nm using a microplate reader, Thermo LUX PH5404 (Thermo Scientific, Waltham, MA, USA). Subsequently, the culture medium was carefully removed, and the wells were gently washed three times with 200 μL of phosphate-buffered saline (PBS) to remove non-adherent cells. The plates were then dried at 37 °C for 30 min and stained with 200 μL of 0.1% (*w*/*v*) crystal violet solution in water for 30 min. Following staining, excess dye was rinsed off with three washes of 200 μL PBS, and the bound dye was solubilized with 200 μL of dimethyl sulfoxide (DMSO) for 1 h. The absorbance of the extracted dye was measured at 570 nm to quantify the extent of biofilm formation. The percentage of biofilm formation was determined through Equation (1).

% Biofilm formation = (OD sample/OD negative control) × 100(1)

### 2.6. Removal of Established C. acnes Biofilms

To degrade mature biofilms, *C. acnes* DMST 14916 suspensions were pre-incubated at 37 °C for 96 h in BHIB_glu_ under an anaerobic environment and static conditions. Non-adherent cells were then removed by washing twice with PBS, and extracts (concentrations ranged from 0.25 × MIC to MIC) were used to treat mature biofilms for 24 h. Biofilms treated with solvent (DMSO) and vancomycin served as negative and positive controls. After treatment, the remaining floating cells were removed, and the biofilm was washed twice with PBS. Biofilm biomass was quantified by adding 100 μL of 0.1% crystal violet to each well for 30 min at room temperature. Excess crystal violet was removed and then dissolved in 99% DMSO for 1 h at room temperature. Absorbance values at 570 nm were obtained using a microplate reader (Thermo LUX PH5404) (Thermo Scientific, Waltham, MA, USA) [25]. The percentage of biofilm formation was determined through Equation (1).

### 2.7. Statistical Analysis

All experiments were performed in triplicate. The data from each experiment represent the mean (SD) of three biological replicates. The ordinary one-way ANOVA and Dunnett’s multiple comparison test was performed using GraphPad Prism ver. 11.0.2 (Software GraphPad, San Diego, CA, USA, https://www.graphpad.com/ (accessed on 3 May 2026). Values were considered significant with *p*-value < 0.0001.

### 2.8. Computational Detail

#### 2.8.1. Protein and Ligand Preparation

The crystal structure of *C. acnes* lipase (_CA_Lipase open form) was selected as the target protein (PDB ID: 6KHM) from the Protein Data Bank (https://www.rcsb.org/structure/6KHM) (accessed on 1 April 2026). The standard amino acid residue was prepared and protonated at pH 7.0 via the H++ web server (http://newbiophysics.cs.vt.edu/H++/) (accessed on 1 April 2026). Meanwhile, the small ligand from fatty acid (FA) compounds was selected as a modeled ligand according to the major presence of total content (>10%) from GLC-MS analysis for DH-W-F-H, DH-W-F-E, and DH-W-D-E extracts, including n-hexadecanoic acid (FA1), linoleic acid ethyl ester (FA2), and hexadecanoic acid ethyl ester (FA3). The geometry of the modeled compounds from the extract was optimized using the Semi-empirical Quantum Austin Model-1 (SQM-AM1) in the Gaussian 09W package [26]. In this work, vancomycin (Van) was used as a control. Continuing, the antechamber [27] and Amber FF14SB force field [28] were used to calculate several parameters for the ligand and protein, such as restrained charge, bonded, and nonbonded, for molecular docking preparation.

#### 2.8.2. Molecular Docking

To obtain the initial conformations of the modeled compounds for the _CA_Lipase binding pocket, molecular docking was performed using the Dock6 software in a Linux environment [29]. In detail, crucial parameters were prepared for grid-box preparation to determine the binding pocket coordinate, such as *grid-spacing* (0.3 Å), center (x: −16.97, y: −52.57, z: −21.29), and dimensions (x: 50.00, y: 35.00, and z: 25.00). The grid score (GS), along with van der Waals (E_vdW_) and electrostatic (E_ele_) components through the *anchor-and-growth* algorithm [30] was calculated to define the interaction energy of protein–ligand complex. The obtained conformation from this step will be used as the initial conformation for further evaluation through molecular dynamics (MD) simulation.

#### 2.8.3. Molecular Dynamics Simulation

The docked conformation was used as a starting point to evaluate the dynamic behavior of the system through MD simulations using the Amber 24 and Ambertools 24 packages [31]. In the topology system preparation, several parameters were applied to prepare the system, including solvation with a TIP3P water box (12 Å of distance) and the addition of sodium ions (Na^+^) to neutralize the system through the tleap tool [32]. The generated system was minimized using the steepest descent method for 1500 steps, followed by the conjugate gradient method for 500 steps for (i) the water and counterion system, (ii) the ligand and protein, and (iii) the whole system. The minimized system was heated gradually from 10 K to 310 K over 200 ps in the heating step. Noted that the temperature was controlled using a Langevin thermostat, while pressure coupling was maintained with a Berendsen barostat. The SHAKE algorithm was used along with a 2 fs integration timestep to constrain all bonds involving hydrogen atoms. The equilibration step was performed for 1300 ps with a stepwise reduction of the harmonic constraints by 30, 20, 10, and 5 kcal/mol/Å^2^. Then, the trajectories can be produced along simulation steps for 200 ns under NPT ensemble conditions (1 atm and 310 K) for 300 ns with the GPU-accelerated [33].

#### 2.8.4. Trajectory Analysis

The generated trajectories for 200 ns were analyzed to evaluate several variables, such as root-mean-squared deviation pairwise (2D-RMSD), superposition, total energy, the root-mean-square deviation (RMSD), radius of gyration (RoG), average structure, and root-mean-square fluctuation (RMSF), by using *cpptraj* and *process_mdout.perl* tools [31,34]. Furthermore, free-energy landscape (FEL), free-energy binding (∆G_bind_), and energy decomposition (${∆G}_{\mathrm{bind}}^{\mathrm{residue}}$) were analyzed using the last 50 ns of trajectories. In particular, the FEL was performed using Bio3D (Version 2.4-5) in the *R*Studio environment [35]. Meanwhile, ∆G_bind_ and (${∆G}_{\mathrm{bind}}^{\mathrm{residue}}$) were estimated using the Molecular Mechanics-Generalized Born Surface Area (MM-GBSA) method with the *MMPBSA.py* tool in AmberTools24 [36].

## 3. Results

### 3.1. D. hispida Extraction

Preliminary phytochemical screening of the water-washed fresh yam using seven standard alkaloid detection reagents (Dragendorff’s, Marmé’s, Mayer’s, Sonnenschein’s, Wagner’s, tannic acid, and Hager’s) showed negative results in all tests, indicating no detectable nitrogen-containing secondary metabolites in the water-washed fresh yam. The reflux extraction results indicated that dried yam samples extracted with 80% ethanol produced the highest extraction yields, reaching 1.39% and 1.80% (Table 1). As expected, extraction of fresh yam samples resulted in lower yields, ranging from 0.51% to 0.97%. Under all experimental conditions, hexane consistently yielded lower extraction efficiencies compared with ethanol. Furthermore, no significant differences in extraction yield were observed between yam samples after dioscorine removal by washing and those that were processed without washing.

### 3.2. Phytochemical Compounds

Extracts were analyzed by capillary GLC/MS. As only volatile substances can be detected by this method, polar non-volatile compounds, such as phenolics, in the extracts might have been overlooked. As shown in Table 2, linoleic acid ethyl ester was identified as the predominant compound in the DH-W-F-E, DH-W-D-E, and DH-NW-D-E extracts. In contrast, stigmasta-5,22-dien-3-ol (stigmasterol) was the major constituent detected in the DH-W-D-H, DH-NW-F-H, and DH-NW-D-H extracts. The DH-W-F-H extract was characterized by a high abundance of n-hexadecanoic acid, whereas 9,12-octadecadienoic acid (Z,Z)- was the principal compound identified in the DH-NW-F-E extract. In addition to these major constituents, other compounds were tentatively detected at concentrations exceeding 1% of the total composition in the yam extracts. Notably, the extract prepared from fresh yam samples using hexane as the extraction solvent exhibited relatively low concentrations of these compounds. Among the detected constituents, 1-methyl-3,4-dihydroisoquinoline is especially significant as a member of the isoquinoline alkaloid family. This compound was present only in the non-water-washed sample.

### 3.3. Antibacterial Assay

The antimicrobial activities of yam extracts at a concentration of 2 mg/mL are summarized in Table 3. Among all tested samples, three extracts, such as DH-W-F-H, DH-W-F-E, and DH-W-D-E, demonstrated notable antibacterial effects, exhibiting activities comparable to the reference antibiotic against all bacterial strains evaluated. These extracts were subsequently selected for microdilution assays to determine their minimum inhibitory concentrations (MICs) and minimum bactericidal concentrations (MBCs), as presented in Table 4 and Figure S1. The MIC values ranged from 64 to 2048 µg/mL, whereas the MBC values ranged from 512 to >2048 µg/mL. DH-W-F-H exhibited the strongest antibacterial activity against *S. aureus* ATCC 25923, with an MIC of 64 µg/mL; however, its MBC exceeded 2048 µg/mL. In addition, *C. acnes* DMST 14916 was most susceptible to DH-W-F-H and DH-W-D-E, both of which exhibited identical MIC and MBC values of 512 µg/mL.

### 3.4. Biofilm Formation Assay

#### 3.4.1. Effect of *D. hispida* Extracts on Bacterial Growth During Anti-Biofilm Assays

**Figure 3:**
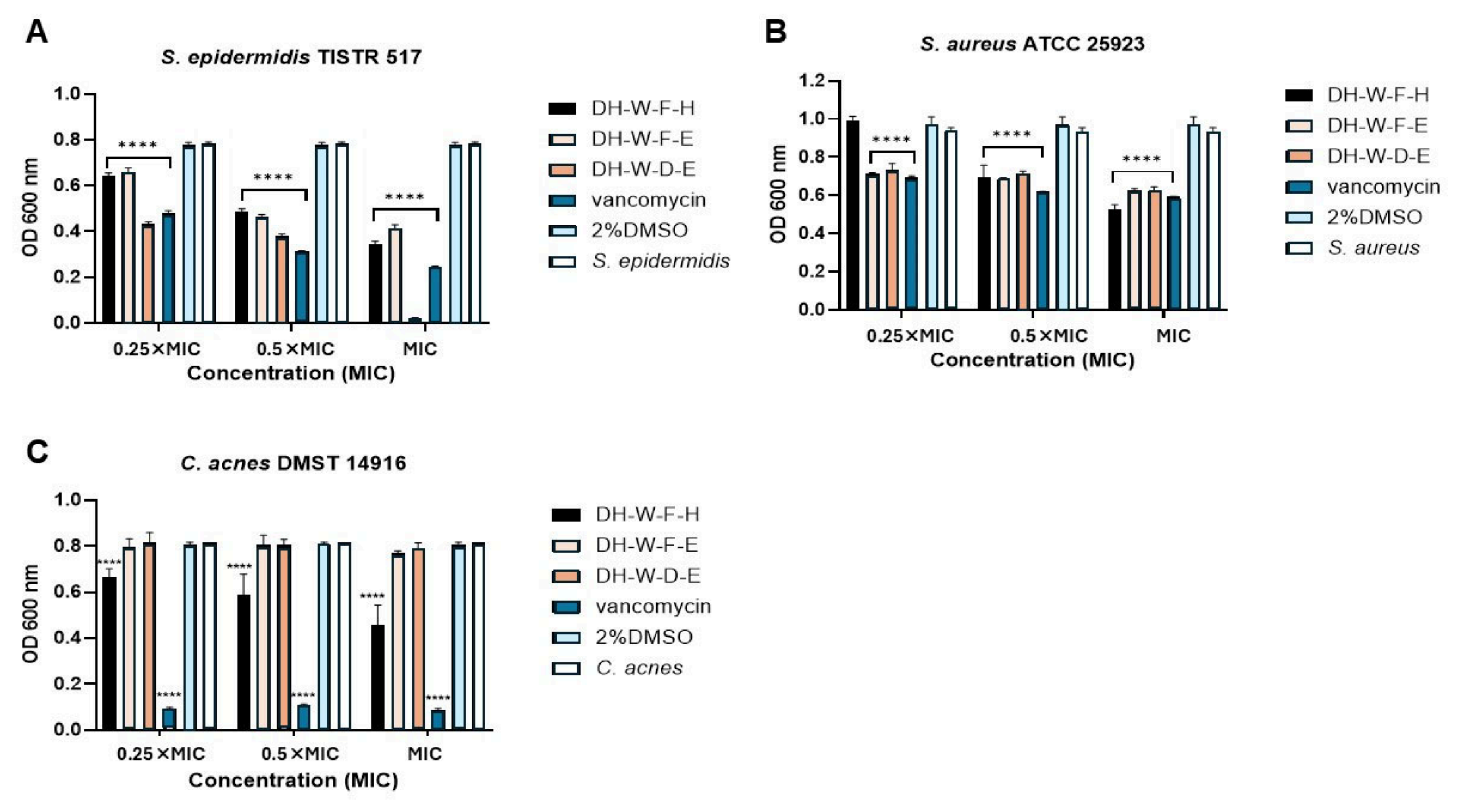
Effect of *D. hispida* extracts on bacterial growth during anti-biofilm assays. Bacterial growth was determined by measuring optical density at 600 nm (OD_600_) following treatment with *D. hispida* extracts DH-W-F-H, DH-W-F-E, and DH-W-D-E at sub-inhibitory and inhibitory concentrations (0.25 × MIC, 0.5 × MIC, and MIC). (**A**) *S. epidermidis* TISTR 517, (**B**) *S. aureus* ATCC 25923, and (**C**) *C. acnes* DMST 14916. Vancomycin was used as the positive control, while 2% DMSO and untreated bacterial cultures served as negative and growth controls, respectively. Results are expressed as mean ± standard deviation. Significant differences compared with the untreated control are indicated by asterisks (**** *p* < 0.0001).

To determine whether the anti-biofilm activity of yam extracts was associated with inhibition of bacterial growth, planktonic cell growth was evaluated by measuring OD_600_ after treatment with DH-W-F-H, DH-W-F-E, and DH-W-D-E at 0.25 × MIC, 0.5 × MIC, and MIC against *S. epidermidis* TISTR 517, *S. aureus* ATCC 25923, and *C. acnes* DMST 14916 (Figure 3). For *S. epidermidis*, all yam extracts reduced bacterial growth in a concentration-dependent manner. DH-W-D-E exhibited the strongest inhibitory effect, with OD_600_ values decreasing from approximately 0.47 at 0.25 × MIC (512 µg/mL) to 0.24 at (2048 µg/mL) MIC. DH-W-F-H and DH-W-F-E also significantly reduced growth compared with the untreated control (*p* < 0.0001), although their effects were less pronounced than those of DH-W-D-E. Vancomycin showed the strongest antibacterial activity, resulting in nearly complete growth inhibition at MIC (0.125 µg/mL). For *S. aureus*, the yam extracts produced a moderate reduction in bacterial growth compared with the untreated control. OD_600_ values decreased slightly as the concentration increased, with DH-W-F-H showing the greatest effect among the extracts. However, bacterial growth remained substantially higher than that observed with vancomycin treatment, indicating limited antibacterial activity against planktonic *S. aureus* cells. For *C. acnes*, DH-W-F-H demonstrated the most pronounced growth inhibition, reducing OD_600_ values from approximately 0.66 at 0.25 × MIC (128 µg/mL) to 0.45 at MIC (512 µg/mL). In contrast, DH-W-F-E and DH-W-D-E exhibited minimal effects on bacterial growth, with OD_600_ values remaining similar to those of the untreated control. Vancomycin markedly suppressed bacterial growth at all tested concentrations (*p* < 0.0001).

#### 3.4.2. Biofilm Inhibition of *D. hispida* Extracts

**Figure 4:**
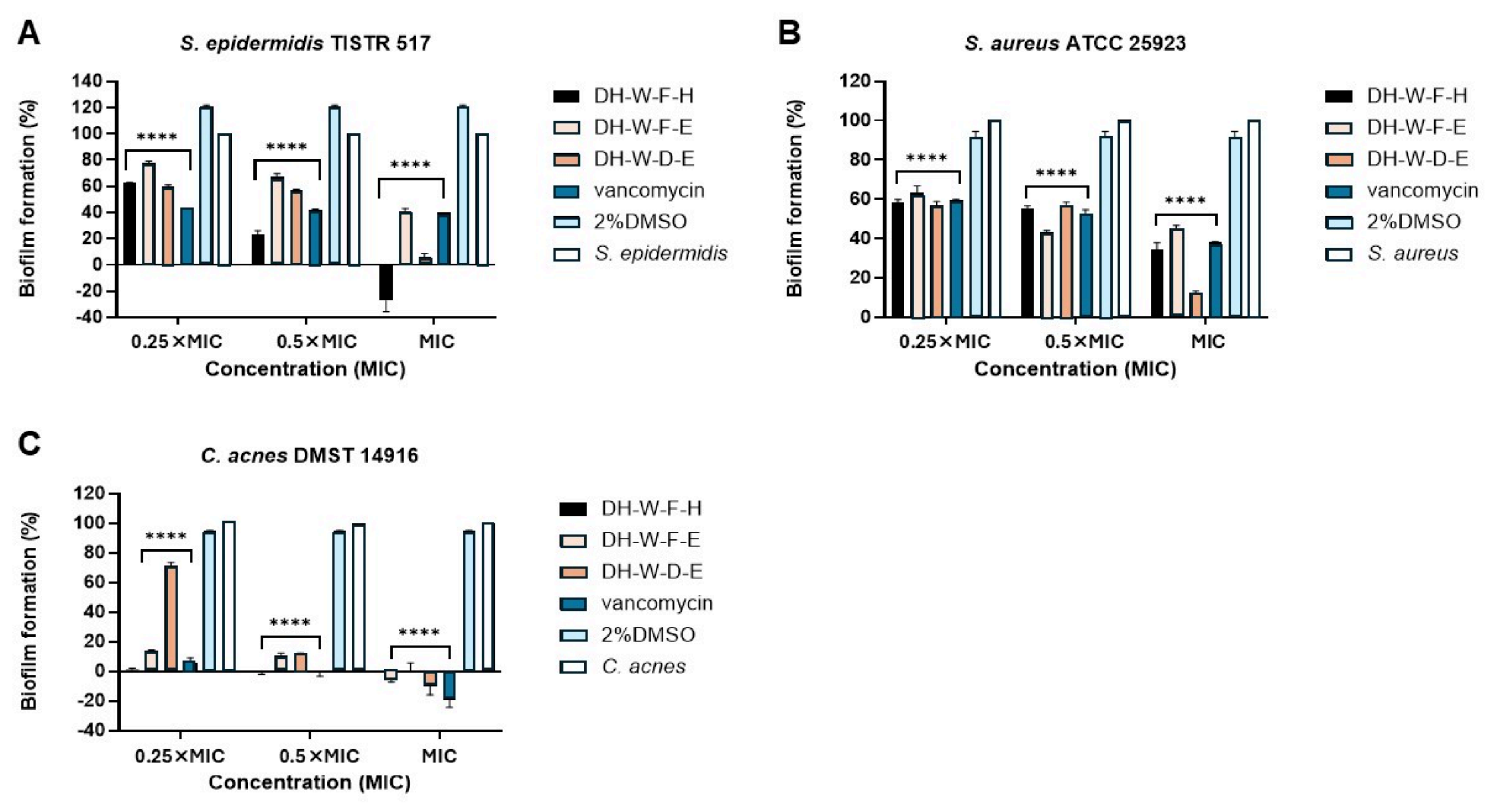
Effects of yam extracts on biofilm formation. Biofilm formation by (**A**) *S. epidermidis* TISTR 517, (**B**) *S. aureus* ATCC 25923, and (**C**) *C. acnes* DMST 14916 following treatments with DH-W-F-H, DH-W-F-E, and DH-W-D-E at 0.25 × MIC, 0.5 × MIC, and MIC concentrations. Vancomycin was used as the positive control, while 2% DMSO and untreated bacterial cultures served as negative and growth controls, respectively. Biofilm formation was expressed as a percentage relative to the untreated control. Data are presented as mean ± SD from three independent experiments. Statistical significance was determined by one-way ANOVA followed by Dunnett’s post hoc test. Asterisks indicate significant differences between treated and control groups (**** *p* < 0.0001).

The anti-biofilm activities of DH-W-F-H, DH-W-F-E, and DH-W-D-E against *S. epidermidis* TISTR 517, *S. aureus* ATCC 25923, and *C. acnes* DMST 14916 are shown in Figure 4. All extracts exhibited concentration-dependent inhibition of biofilm formation, with significant differences compared to the controls at all tested concentrations (***, *p* < 0.0001). Against *S. epidermidis*, DH-W-F-H demonstrated the strongest activity, reducing biofilm formation from approximately 78% at 0.25 × MIC (512 µg/mL) to −25% at MIC (2048 µg/mL), indicating nearly complete biofilm eradication, while DH-W-F-E and DH-W-D-E showed moderate inhibitory effects. Similarly, all extracts significantly inhibited biofilm formation by *S. aureus*, with DH-W-F-H exhibiting the greatest reduction at MIC, followed by DH-W-F-E and DH-W-D-E, showing activities comparable to or greater than vancomycin. The most pronounced anti-biofilm effects were observed against *C. acnes*, where all extracts markedly reduced biofilm formation at sub-MIC concentrations and nearly abolished biofilm development at MIC, resulting in negative biofilm percentages. In contrast, the untreated and 2% DMSO controls consistently maintained high biofilm formation levels, confirming that the observed inhibitory effects were attributable to the extracts. Overall, *C. acnes* was the most susceptible strain, followed by *S. epidermidis* and *S. aureus*, highlighting the potent anti-biofilm potential of the tested extracts.

### 3.5. Biofilm Degradation

**Figure 5:**
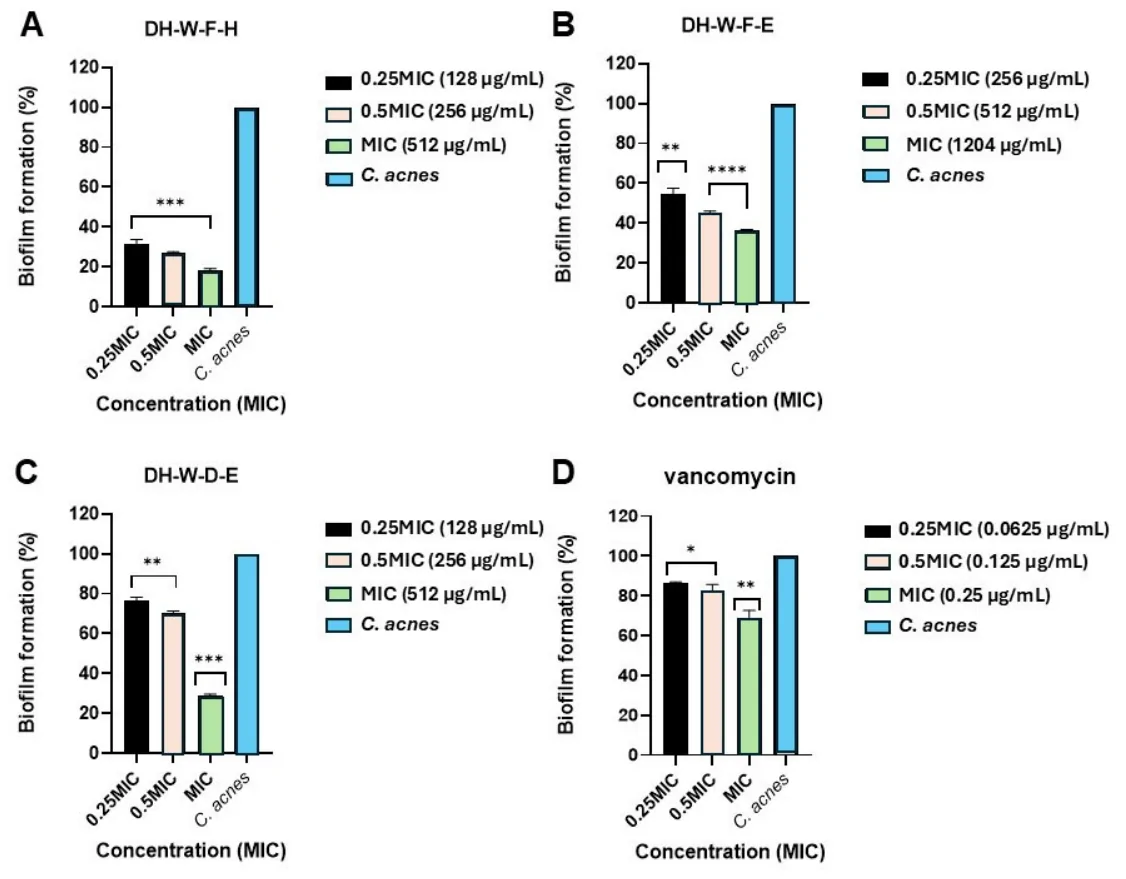
Effects of *D. hispida* extracts and vancomycin on established biofilms of *C. acnes* DMST 14916. Established biofilms of *C. acnes* were treated with (**A**) DH-W-F-H, (**B**) DH-W-F-E, (**C**) DH-W-D-E, and (**D**) vancomycin at 0.25 × MIC, 0.5 × MIC, and MIC concentrations. Residual biofilm formation was quantified and expressed as a percentage relative to the untreated control (*C. acnes*), which was set at 100%. Data are presented as mean ± SD from three independent experiments. Statistical significance between treatment concentrations was determined using one-way ANOVA followed by Dunnett’s post hoc test. Asterisks indicate significant differences (* *p* < 0.05, ** *p* < 0.01, *** *p* < 0.001, **** *p* < 0.0001).

The effects of DH-W-F-H, DH-W-F-E, DH-W-D-E, and vancomycin on established biofilms of *C. acnes* DMST 14916 are shown in Figure 5. All treatments significantly reduced established biofilm formation in a concentration-dependent manner compared with the untreated control, which maintained approximately 100% biofilm formation. Among the tested extracts, DH-W-F-H exhibited the strongest biofilm eradication activity, reducing the remaining biofilm to approximately 31%, 26%, and 17% at 0.25 × MIC (128 µg/mL), 0.5 × MIC (256 µg/mL), and MIC (512 µg/mL), respectively. Similarly, DH-W-F-E reduced established biofilm formation to approximately 55%, 44%, and 35%, while DH-W-D-E reduced biofilm formation to approximately 76%, 69%, and 28% at the respective concentrations. In contrast, vancomycin showed comparatively lower efficacy, with substantial biofilm remaining at approximately 86%, 81%, and 67% following treatment at 0.25 × MIC (0.0625 µg/mL), 0.5 × MIC (0.125 µg/mL) and MIC (0.25 µg/mL), respectively. Statistical analysis revealed significant reductions in established biofilm formation with increasing concentrations of all treatments.

### 3.6. Molecular Docking Studies

**Figure 6:**
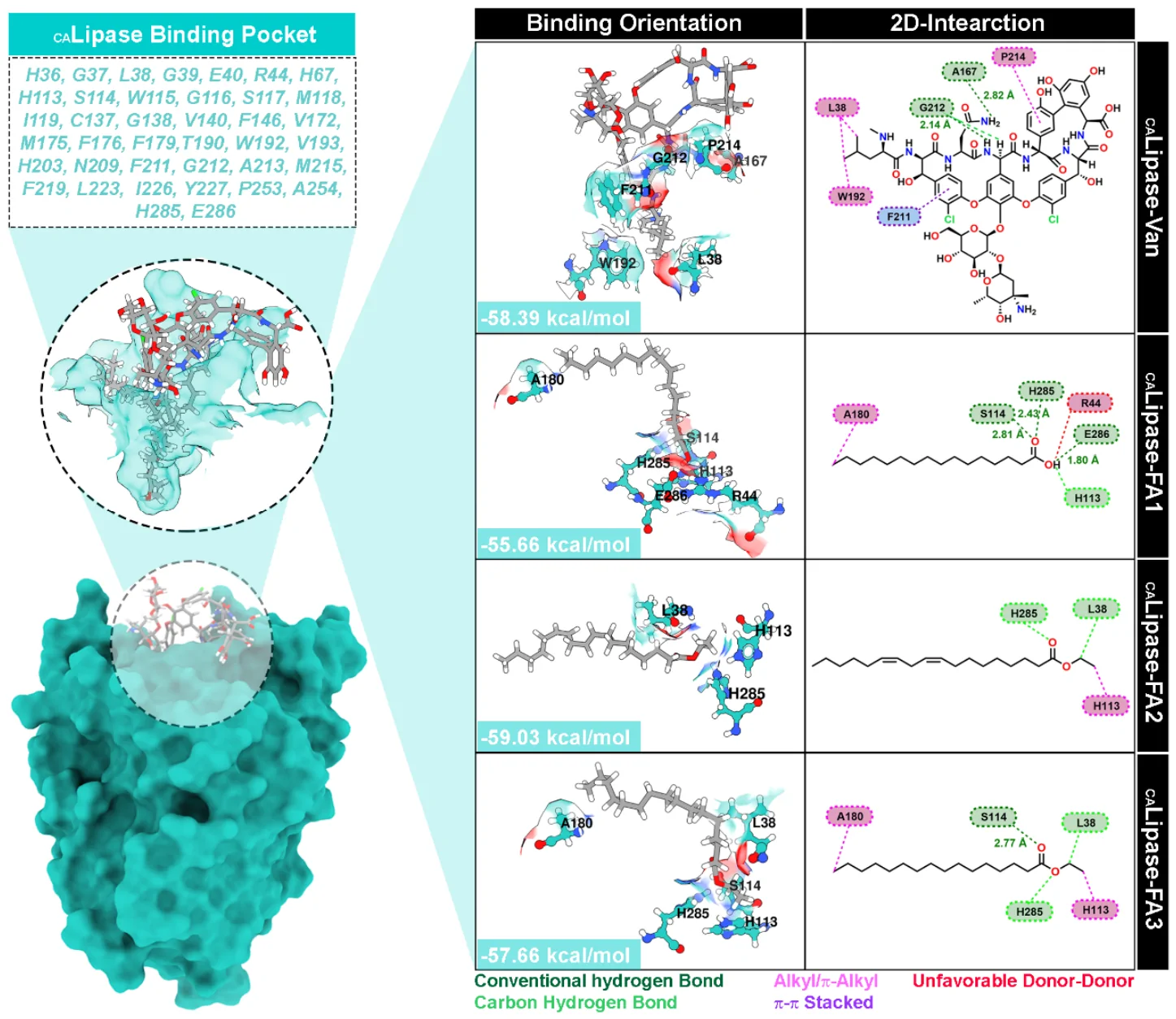
The _CA_Lipase–ligand interaction is presented by grid score (GS), binding orientation, and 2D interaction types.

Molecular docking studies were carried out to examine the binding interactions of the major fatty acid derivatives identified in the DH-W-F-H, DH-W-F-E, and DH-W-D-E extracts (Table 2), namely n-hexadecanoic acid (FA1), linoleic acid ethyl ester (FA2), and hexadecanoic acid ethyl ester (FA3), with the *C. acnes* lipase protein (_CA_Lipase). The protein structure was obtained from the Protein Data Bank (PDB ID: 6KHM) based on its suitable resolution and well-defined binding pocket (Figure 6). Vancomycin (Van) was included as a reference compound for comparative analysis. Based on binding pocket visualization, the amino acid residues involved in the _CA_Lipase binding pocket indicate a relatively broad active pocket dominated by polar and hydrophobic residues. The interaction between the ligand and the protein revealed that the control (Van) had a binding energy of −58.39 kcal/mol, with conventional hydrogen bond formation at key residues such as A167 (2.82 Å) and G212 (2.14 Å), as well as additional interactions in the form of alkyl/π-alkyl and π-π stackING. This indicates strong affinity but high structural complexity, which may affect penetration and efficiency against biofilm targets. This is proven by the bulky structure of Van because it cannot penetrate deeper into the binding pocket compared to FA derivatives. Interestingly, FA derivatives (FA1, FA2, and FA3) exhibited comparable binding energies of −55.66, −59.03, and −57.66 kcal/mol, respectively (Table 5). FA2 specifically demonstrated the highest affinity, presumably due to its optimal orientation (planar conformation) within the binding pocket and stable hydrogen interactions with residues such as L38, H113, and H285. Furthermore, hydrophobic interactions along the aliphatic chain of FA allow for better adaptation to the nonpolar environment within the deeper binding pocket, a characteristic lacking in the more bulky/complex and polar vancomycin. These findings suggest lipid-based compounds have an advantage in targeting enzymes involved in lipid metabolism of *C. acne*.

### 3.7. Equilibrium and Dynamic Behaviors

**Figure 7:**
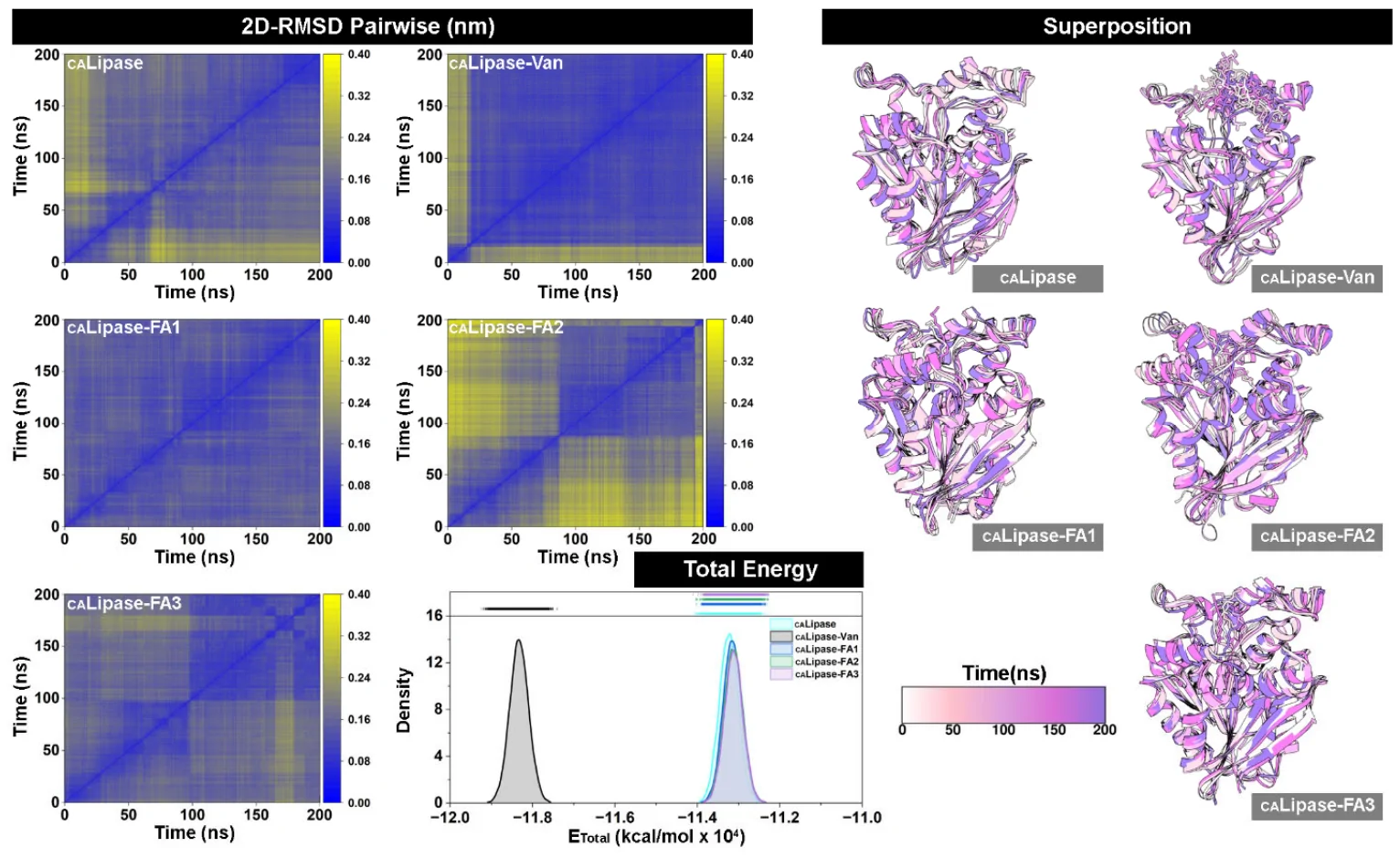
The system equilibrium was assessed by root-mean-squared deviation pairwise (2D-RMSD), superposition, and total energy along 200 ns of simulation trajectories. In particular, the superposition was extracted with different conformations according to the time change during the simulation, which ranged from 0 ns (white) to 200 ns (purple).

**Figure 8:**
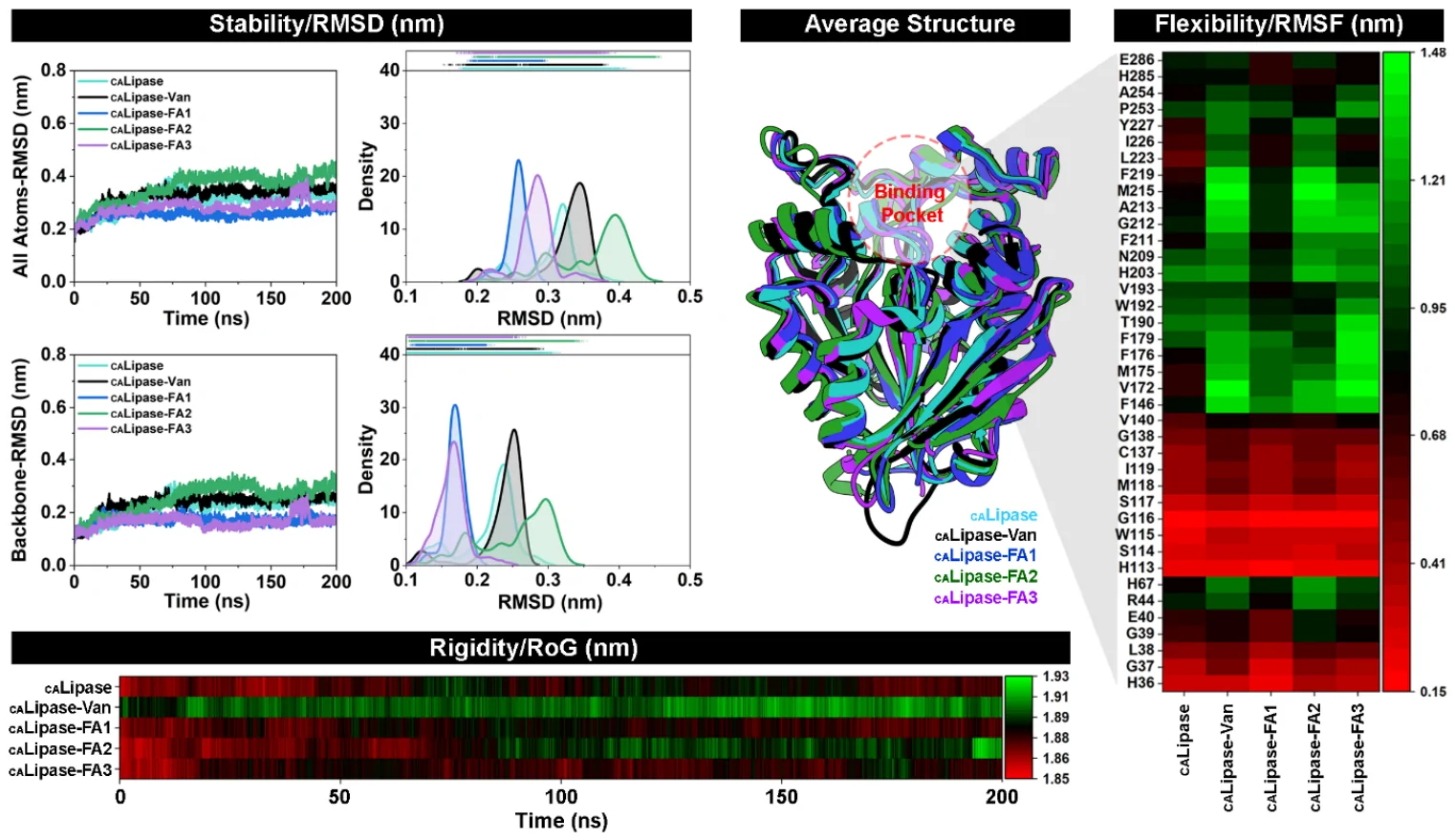
The root-mean-squared deviation (RMSD), average structure, root-mean-squared fluctuation (RMSF), and radius of gyration (RoG) are plotted over 200 ns of trajectories using the *cpptraj* tool. The RMSD distribution is plotted using kernel smoothing. Specifically, several amino acids in the binding pocket were visualized to show their flexibility through RMSF according to the heatmap coloring from the lowest (red) to the highest (green). Moreover, the RoG (nm) split heatmap is colored from the lowest (red) to the highest (green).

The equilibrium and dynamic behavior of _CA_Lipase in both Apo and ligand-bound states were evaluated using 200 ns molecular dynamics simulations (Figure 7 and Figure 8). Pairwise 2D-RMSD analyses were performed to assess conformational convergence throughout the simulation trajectory based on Cα atom movements. The Apo _CA_Lipase system exhibited broader RMSD variations during the early and middle stages of the simulation, indicating structural rearrangements before reaching equilibrium. In contrast, the _CA_Lipase–Van and _CA_Lipase–FA1 complexes displayed more homogeneous RMSD distributions characterized by predominant low-deviation regions, suggesting earlier conformational stabilization. The _CA_Lipase–FA2 complex showed slightly elevated RMSD values during the initial simulation period but reached a stable conformational state after approximately 100 ns. Similar stabilization behavior was observed for the _CA_Lipase–FA3 complex. Structural superposition analyses further demonstrated that all systems retained their overall protein architecture throughout the simulation period without significant conformational deviations. However, the ligand-bound complexes exhibited greater structural compactness than the Apo protein. Among them, the _CA_Lipase–FA2 and _CA_Lipase–FA3 complexes showed the highest degree of structural overlap, particularly around the binding region.

The total energy profiles were consistent with the structural analyses. The Apo system and the FA1-, FA2-, and FA3-bound complexes exhibited overlapping and relatively narrow energy distributions centered around approximately −11.36 × 10^4^ kcal/mol, indicating similar energetic behavior and convergence. In contrast, the _CA_Lipase–Van complex displayed a distinct energy distribution centered around approximately −11.86 × 10^4^ kcal/mol. Despite this shift, the energy profile remained stable throughout the simulation. Collectively, the 2D-RMSD, structural superposition, and total energy analyses indicate that all systems achieved equilibrium and maintained stable conformations over the 200 ns simulation period.

Quantitative molecular dynamics analyses were performed using root mean square deviation (RMSD), root mean square fluctuation (RMSF), and radius of gyration (Rg) parameters to evaluate the stability of _CA_Lipase and its ligand-bound complexes during the 200 ns simulation. The RMSD trajectories, including both all-atom and backbone atoms (Cα, C, N, and O), showed that all systems reached equilibrium after approximately 20–40 ns. The ligand-bound complexes exhibited backbone RMSD values ranging from approximately 0.15 to 0.35 nm, which were generally lower than those observed for the apo protein. Kernel density estimation of RMSD values revealed narrow distribution peaks for all systems, indicating the presence of stable conformational populations throughout the simulation. RMSF analysis demonstrated that most amino acid residues exhibited low to moderate fluctuations. Residues located within or adjacent to the binding pocket displayed slightly elevated fluctuations in the ligand-bound systems compared with the Apo protein. This behavior was particularly evident in the fatty acid derivative complexes, where localized flexibility was observed around the binding region while the overall protein structure remained stable. The radius of gyration analysis showed minimal variation throughout the simulation period for all systems. Comparable Rg values were maintained during the 200 ns trajectory, indicating preservation of overall structural compactness regardless of ligand binding. Collectively, the RMSD, RMSF, and Rg analyses suggest that both the apo and ligand-bound systems maintained stable conformations during the simulation period.

### 3.8. Free-Energy Landscape

**Figure 9:**
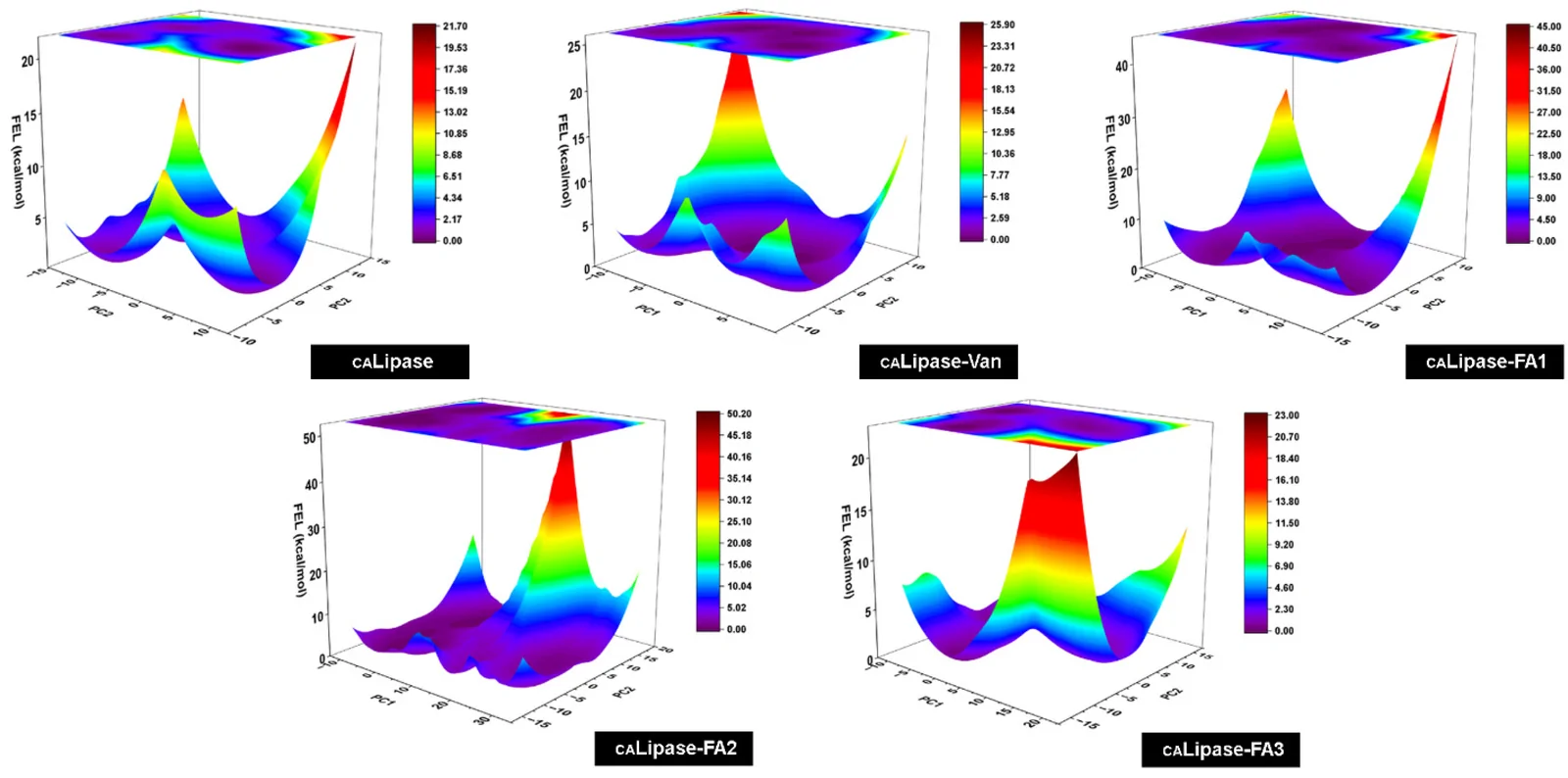
Free-energy landscape (FEL) was extracted from Cα of amino acid residues by using the last 150–200 ns of trajectories. The FEL was generated by projecting the first two principal components (PC1 and PC2).

Following confirmation of system equilibration over the 200 ns simulation period (Figure 7), free-energy landscape (FEL) analysis was performed using the final 50 ns trajectory segment (150–200 ns) to characterize the conformational states sampled by each system (Figure 9). The FELs were constructed by projecting the simulation trajectories onto the first two principal components (PC1 and PC2). Distinct energy landscape profiles were observed for each system. The Apo _CA_Lipase system exhibited multiple well-separated low-energy basins distributed across conformational space. These energy minima suggest the existence of several accessible conformational states during the simulation. In contrast, the ligand-bound systems displayed more localized energy minima with reduced dispersion across the principal component space, indicating a more restricted conformational distribution. The observed FEL profiles were consistent with the RMSD and 2D-RMSD analyses (Figure 7 and Figure 8), which showed greater conformational variability in the Apo system compared with the ligand-bound complexes. Overall, the results indicate that ligand binding influenced the conformational sampling behavior of _CA_Lipase during the simulation period.

In contrast, the complex system, particularly _CA_Lipase-FA2 and _CA_Lipase-FA3, exhibits a more focused energy landscape with a deeper and well-defined global minimum. This indicates that the system is trapped in a dominant, thermodynamically stable conformational state. The _CA_Lipase-FA2 complex exhibits the deepest and narrowest energy well, indicating the highest stability and low conformational heterogeneity. Meanwhile, _CA_Lipase-FA1 and _CA_Lipase-Van still exhibited several local minima, reflecting the presence of conformations despite remaining stable. Overall, these FEL results corroborate findings from previous dynamics analyses that binding of fatty acid derivatives, particularly FA2, can stabilize proteins more effectively by limiting the accessible conformational space during simulations.

### 3.9. Free-Energy Binding and Decomposition Energy

**Figure 10:**
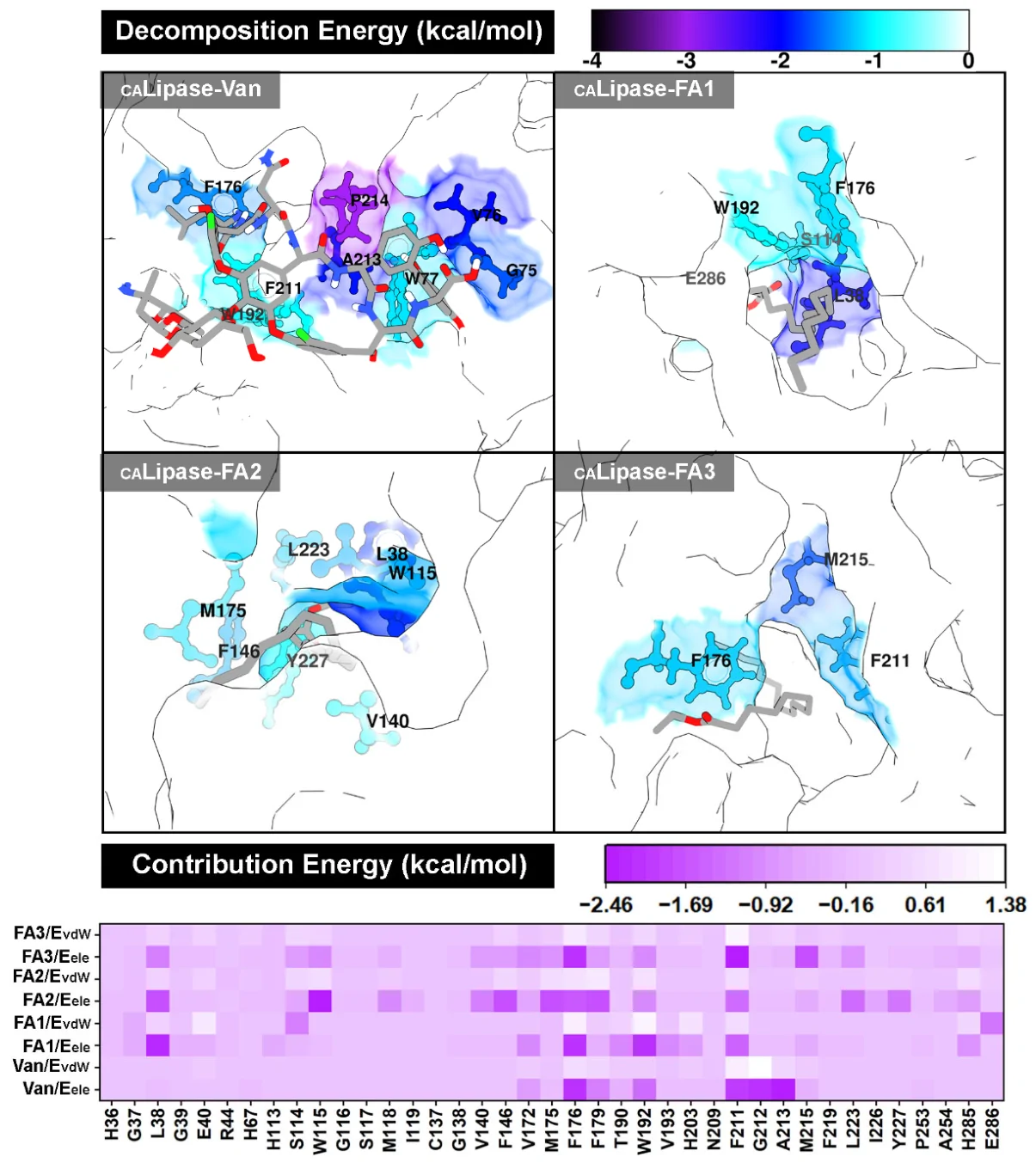
Energy decomposition (${∆G}_{\mathrm{bind}}^{\mathrm{residue}}$) and contribution energy (CE) were calculated using the MM-GBSA approach with the last 150–200 ns of trajectories. Noted that contribution energy was defined by the contribution E_vdw_ (ΔE_vdW_ + ${∆G}_{\mathrm{solv}}^{\mathrm{nonpolar}}$) and E_ele_ (ΔE_ele_ + ${∆G}_{\mathrm{solv}}^{\mathrm{GB}}$), which is described by heatmap coloring from the weaker (white) to the stronger (purple).

Free-energy landscape (FEL) analysis identified the minimum-energy conformations sampled by each system during the final 50 ns of the simulation trajectory (Figure 9). Based on these equilibrated conformations, binding free-energy (ΔG_bind_) and decomposition energy (${∆G}_{\mathrm{bind}}^{\mathrm{residue}}$) analyses were performed using the MM-GBSA method. The calculated binding free energies and individual energy components are summarized in Table 6. All protein–ligand complexes exhibited negative ΔG_bind_ values. Among the evaluated systems, the _CA_Lipase–FA2 complex showed the most favorable binding free energy (−38.05 ± 0.16 kcal/mol), followed by _CA_Lipase–FA1 (−35.33 ± 0.19 kcal/mol), _CA_Lipase–FA3 (−31.93 ± 0.16 kcal/mol), and _CA_Lipase–Van (−26.73 ± 0.21 kcal/mol). The energy decomposition analysis indicated that van der Waals interactions constituted a major energetic contribution in the fatty acid derivative complexes. In contrast, the vancomycin complex exhibited substantial electrostatic and nonpolar solvation contributions, accompanied by a larger polar solvation term. Per-residue energy decomposition analysis (Figure 10) identified several binding-pocket residues with energy contributions ${∆G}_{\mathrm{bind}}^{\mathrm{residue}}$ lower than −1.00 kcal/mol. These residues consistently contributed to ligand stabilization throughout the simulation trajectories. The contribution energy maps revealed distinct distributions of van der Waals-related energy terms (ΔE_vdW_ + ${∆G}_{\mathrm{solv}}^{\mathrm{nonpolar}}$) and electrostatic-related energy terms (ΔE_ele_ + ${∆G}_{\mathrm{solv}}^{\mathrm{GB}}$) among the investigated complexes. Collectively, these results demonstrate persistent interactions between the ligands and the _CA_Lipase binding pocket over the simulation period. 

These computational findings strongly align with in vitro data from extracts, which were reported to exhibit superior anti-biofilm and antibacterial activity compared to vancomycin as a control. This biological advantage can be explained mechanistically at the molecular level through MM-GBSA and docking results. Fatty acid compounds, particularly FA2, exhibited the highest binding affinity and optimal interaction stability with _CA_Lipase’s binding pocket. The dominance of hydrophobic interactions localized to key residues allows FA2 to bind more effectively within the lipophilic pocket site, potentially inhibiting enzyme function more efficiently. Thus, the correlation between in vitro activity and in silico results may lead to the hypothesis that the active compounds in the extract (DH-W-F-H, DH-W-F-E, and DH-W-D-E), particularly those resembling the FA2 structure, contribute significantly to *C. acnes* biofilm inhibition through the _CA_Lipase inhibition mechanism.

## 4. Discussion

*D. hispida* is a yam species traditionally consumed after detoxification and widely recognized as a source of bioactive metabolites with potential pharmacological applications. Previous studies have reported the occurrence of alkaloids, cyanogenic glucosides, phenolics, flavonoids, saponins, terpenoids, and fatty acid derivatives in *Dioscorea* species, many of which exhibit antimicrobial, antioxidant, and anti-inflammatory activities [18]. Fatty acid esters have also been reported to possess antimicrobial properties, although their activity depends strongly on the chemical structure and ester moiety [37]. Compounds such as β-sitosterol, stigmasterol, and campesterol have received considerable attention because of their broad biological properties. Similarly, terpenoids represent a structurally diverse group of plant metabolites with reported antimicrobial, antioxidant, and anti-inflammatory activities. Their biological effects have been associated with interactions with cellular membranes, modulation of oxidative stress, and regulation of inflammatory pathways [38].

In the present study, extraction yield and chemical composition were strongly influenced by sample pretreatment and solvent selection. Dried yam samples extracted with 80% ethanol produced significantly higher extraction yields than fresh samples. Differences in extraction yield may be attributed to variations in the moisture content of the starting material as well as the degree of tissue disruption during sample preparation. The water content influences the proportion of extractable dry matter, whereas crushing or chopping increases the exposed surface area and facilitates solvent penetration, thereby affecting extraction efficiency [39]. In addition, moisture removal enhances cell wall disruption, improves solvent penetration, and facilitates mass transfer during extraction [40]. Furthermore, the superior extraction efficiency of aqueous ethanol compared with hexane can be attributed to its intermediate polarity, which enables the recovery of a broader range of phytochemicals, whereas hexane preferentially extracts nonpolar constituents [40]. In contrast, washing had little effect on the overall extraction yield, suggesting that the removal of water-soluble compounds such as dioscorine did not substantially alter the total extractable material. In addition to facilitating the removal of dioscorine, the washing process also contributes to the reduction in cyanogenic glycosides, another group of toxic compounds naturally present in yam tubers. Because cyanogenic glycosides are highly water-soluble, soaking and washing effectively promote their leaching into the surrounding water, thereby lowering their concentration in the plant material. Furthermore, the processing steps employed during yam extract preparation, including peeling, slicing, drying, soaking, and boiling, are consistent with traditional detoxification practices and collectively contribute to reducing cyanogenic glycoside content. In particular, thermal processing further enhances detoxification by promoting the degradation and volatilization of cyanide released from cyanogenic glycosides, resulting in a safer raw material for subsequent extraction [13].

GLC-MS analysis was used in this study to successfully identify a number of volatile and semi-volatile components of the *D. hispida* extract. However, only sufficiently volatile and thermally stable substances can be used with GLC-MS. As a result, the current analysis may not have found many classes of bioactive phytochemicals that have been previously identified in *Dioscorea* species, such as phenolic compounds, flavonoids, steroidal saponins, alkaloids, and other polar secondary metabolites. In order to obtain a more thorough characterization of these non-volatile metabolites and to further clarify the connection between the phytochemical composition and the observed biological activities, future research utilizing LC–MS or LC–MS/MS analysis needs to be conducted.

GLC–MS analysis revealed considerable variation in metabolite composition among extracts. Fatty acids, fatty acid esters, phytosterols, terpenoids, hydrocarbons, and tocopherol derivatives were the major constituents detected. However, since GLC-MS can only detect volatile compounds, our analyses may have overlooked some polar natural products, such as flavonoids and other phenolics.

Washing appeared to influence metabolite recovery, particularly in fresh samples, possibly through the removal of extracellular metabolites and soluble precursor compounds, which can alter the resulting metabolite profile [41]. Drying promoted the accumulation and recovery of phytosterols, including campesterol and stigmasterol, likely due to reduced enzymatic degradation and enhanced accessibility of intracellular lipophilic compounds [42]. The extraction solvent also strongly influenced metabolite classes. Ethanol extracts were dominated by fatty acid ethyl esters, particularly linoleic acid ethyl ester and hexadecanoic acid ethyl ester. Ethanol is known to efficiently solubilize lipid-derived compounds, although esterification and transesterification reactions occurring during extraction may also contribute to the formation of ethyl ester derivatives [43]. In contrast, hexane extracts contained higher proportions of free fatty acids and phytosterols, reflecting the hydrophobic nature of these compounds and their preferential solubility in nonpolar solvents [44,45]. Several major constituents identified in the extracts have previously been associated with biological activities. Fatty acids such as hexadecanoic acid and linoleic acid have been reported to possess antimicrobial properties by disrupting microbial membranes and interfering with cellular metabolism [46]. In addition, phytosterols including campesterol, stigmasterol, and γ-sitosterol are recognized for their antioxidant, anti-inflammatory, and health-promoting effects [47].

The antimicrobial evaluation demonstrated that yam extracts exerted stronger anti-biofilm effects than direct inhibition of planktonic bacterial growth. Although only moderate reductions in OD_600_ values were observed, substantial inhibition of biofilm formation occurred, particularly at MIC concentrations. This discrepancy suggests that the extracts primarily target biofilm-associated processes rather than bacterial viability. Similar effects have been reported for plant-derived metabolites that interfere with bacterial adhesion, quorum sensing, and extracellular polymeric substance (EPS) production, thereby suppressing biofilm development without exerting strong bactericidal activity [48]. Notably, *C. acnes* was the most susceptible microorganism to biofilm inhibition despite relatively limited effects on planktonic growth. Because biofilm formation is a key factor contributing to acne pathogenesis, inflammation, and treatment resistance, these findings suggest potential applications of *D. hispida* extracts as anti-acne agents.

Lipase is recognized as an important virulence factor of *C. acnes* because it hydrolyzes sebum triglycerides into free fatty acids that contribute to follicular inflammation and biofilm development. Consequently, inhibition of lipase has been proposed as a promising strategy for reducing *C. acnes* pathogenicity and acne-associated biofilms [49]. Molecular docking demonstrated that the major fatty acid derivatives identified in the extracts exhibited favorable binding toward _CA_Lipase, with FA2 showing the strongest binding affinity. The interaction was stabilized by hydrogen bonding with key active-site residues and extensive hydrophobic contacts within the lipophilic binding cavity. Compared with vancomycin, the fatty acid derivatives adopted deeper binding conformations, which may be attributed to their smaller molecular size and greater lipophilicity. These findings are consistent with the lipid-rich environment of the enzyme and suggest that fatty acid derivatives are well suited for targeting lipase-associated pathways in *C. acnes*.

To further validate the docking results, molecular dynamics simulations were performed over 200 ns. The ligand-bound complexes displayed lower structural fluctuations than the Apo protein, indicating that ligand binding contributed to _CA_Lipase stabilization. Among the tested compounds, FA2 and FA3 showed the most stable interaction profiles, as evidenced by RMSD, RMSF, and radius of gyration analyses. In addition, free-energy landscape analysis revealed fewer conformational states in the ligand-bound systems, suggesting reduced structural flexibility upon ligand binding. These observations indicate the formation of stable protein–ligand complexes throughout the simulation period [50].

Binding free-energy calculations using the MM-GBSA method further supported the docking and simulation results. All ligands exhibited favorable binding energies, with FA2 showing the most negative ΔG_bind_ value. The binding process was primarily driven by van der Waals interactions, highlighting the importance of hydrophobic contacts between the long aliphatic chains of the fatty acid derivatives and the nonpolar residues lining the _CA_Lipase binding pocket [36,51]. Notably, FA2 exhibited a stronger binding affinity than vancomycin, suggesting that lipid-derived compounds may represent promising inhibitors of _CA_Lipase. This finding should be interpreted in the context of vancomycin’s known limitations as an anti-biofilm agent. Although vancomycin remains a first-line treatment against many Gram-positive pathogens, its efficacy against mature biofilms is often substantially reduced due to limited penetration through the extracellular polymeric matrix, altered physiological states of biofilm-embedded cells, and the presence of antibiotic-tolerant persister populations. Consequently, vancomycin frequently exhibits poor biofilm eradication activity and requires concentrations far exceeding clinically achievable levels to eliminate established biofilms [52,53]. Therefore, while the superior binding affinity of FA2 relative to vancomycin supports its potential as a _CA_Lipase inhibitor, the use of vancomycin as the sole positive control may provide a conservative benchmark for assessing anti-biofilm efficacy. Future studies should compare these compounds with established anti-biofilm agents and validate their inhibitory activity through in vitro and in vivo biofilm models.

The computational findings agree well with the biological assays, in which fatty acid-rich extracts (DH-W-F-H, DH-W-F-E, and DH-W-D-E) exhibited pronounced inhibition of *C. acnes* biofilm formation despite only moderate effects on planktonic growth. Fatty acids have been recognized as natural anti-biofilm agents capable of interfering with bacterial adhesion, cell-surface hydrophobicity, extracellular polymeric substance production, and virulence-associated processes. Kim et al. [48] demonstrated that 17 of 24 saturated and unsaturated fatty acids inhibited *C. acnes* biofilm formation by 60–99% at 20 µg/mL. This observation supports the hypothesis that the extracts primarily interfere with virulence-related processes rather than bacterial viability. Collectively, the results suggest that fatty acid derivatives present in *D. hispida* may contribute to anti-biofilm activity through _CA_Lipase inhibition and highlight their potential as natural anti-acne agents. Given the important role of *C. acnes* biofilms in persistent acne lesions and reduced responsiveness to conventional antimicrobial therapies, these findings may have practical implications for the development of cosmetic and dermatological products. Fatty acid-rich yam extracts could potentially be incorporated into topical formulations, such as anti-acne creams, gels, cleansers, or cosmeceutical products [22], to prevent biofilm formation and reduce bacterial virulence while minimizing the selective pressure associated with bactericidal agents. Furthermore, the anti-biofilm properties of these extracts may have broader medical applications, including their use as natural bioactive ingredients in wound-care formulations or antimicrobial coatings designed to limit biofilm-associated skin infections. Nevertheless, direct enzyme inhibition assays and additional mechanistic studies are required to confirm the proposed mode of action and to evaluate their efficacy and safety in clinical settings.

## 5. Conclusions

This study demonstrated that fatty acid-rich extracts and fractions from *D. hispida* possess promising anti-biofilm activity against *C. acnes*. The extracts effectively inhibited biofilm formation while exhibiting only moderate antibacterial activity against planktonic cells, suggesting a mechanism that primarily targets virulence-related processes rather than bacterial growth. Chemical profiling revealed the presence of fatty acid derivatives, and in silico analyses indicated that these compounds interact favorably with _CA_Lipase, a key virulence factor associated with *C. acnes* pathogenicity. Among the identified compounds, FA2 showed the strongest binding affinity and the most stable protein–ligand interactions throughout molecular dynamics simulations. The agreement between the biological and computational results suggests that fatty acid derivatives from *D. hispida* may contribute to anti-biofilm activity through _CA_Lipase inhibition. These findings highlighted the potential value of *D. hispida* as a source of natural bioactive compounds for the development of functional ingredients in cosmetic, dermatological, and other health-related applications aimed at controlling biofilm-associated skin conditions. Further studies involving direct enzyme inhibition assays, safety assessments, formulation development, and in vivo validation are warranted to confirm their efficacy and practical applicability.

## Figures and Tables

**Table 1:** Yields of *D. hispida* extracts obtained by different solvents and treatments.

Sample Preparation		Solvent Extraction	Code	Sample Weight (g)	Extract Weight (g)	Yield (%)
Washing	Type					
Water washing	Fresh	Hexane (H)	1. DH-W-F-H	1423.18	1.12	0.08
		80% Ethanol (E)	2. DH-W-F-E	1365.44	6.91	0.51
	Dry	Hexane (H)	3. DH-W-D-H	400.14	0.45	0.11
		80% Ethanol (E)	4. DH-W-D-E	407.21	5.68	1.39
Non-water washing	Fresh	Hexane (H)	5. DH-NW-F-H	20,131.68	0.52	0.0026
		80% Ethanol (E)	6. DH-NW-F-E	1783.33	17.38	0.97
	Dry	Hexane (H)	7. DH-NW-D-H	841.71	1.03	0.12
		80% Ethanol (E)	8. DH-NW-D-E	730.26	13.17	1.80

**Table 2:** Tentative identifications of volatile natural products of *D. hispida* extracts.

RT	Compound Name	% of Total							
		DH-W-F-H	DH-W-F-E	DH-W-D-H	DH-W-D-E	DH-NW-F-H	DH-NW-F-E	DH-NW-D-H	DH-NW-D-E
	**Furan derivatives**								
4.39	2-Furanmethanol	2.67							
	**Cyclic ketones/Carbonyl compounds**								
5.32	1,2-Cyclopentanedione	2.83							
	**Lactones**								
6.08	2-Hydroxy-gamma-butyrolactone					1.00			
29.77	4,8,12,16-Tetramethylheptadecan-4-olide		1.43	1.27					
	**Pyranone derivatives**								
8.88	4H-Pyran-4-one, 2,3-dihydro-3,5-dihydroxy-6-methyl-						2.77		
	**Alkaloids/Nitrogen-containing heterocycles**								
11.76	1-Methyl-3,4-dihydroisoquinoline						1.43		2.33
	**Fatty acids (free fatty acids)**								
22.22	n-Hexadecanoic acid (Palmitic acid)	13.36 *					2.90		
26.08	9,12-Octadecadienoic acid (Z,Z)- (Linoleic acid)						6.51 *		
33.15	Erucic acid							1.02	
	**Fatty acid methyl esters (FAMEs)**								
21.05	Hexadecanoic acid, methyl ester			4.23	6.37			1.49	
24.86	9,12-Octadecadienoic acid (Z,Z)-, methyl ester			4.90	5.44			2.38	
25.05	9-Octadecenoic acid (Z)-, methyl ester			1.09	2.54				
25.66	Methyl stearate			1.31	1.80				
	**Fatty acid ethyl esters (FAEEs)**								
22.71	Hexadecanoic acid, ethyl ester		14.23	3.46	8.9		2.84	4.33	5.04
26.30	Linoleic acid ethyl ester		20.05 *	4.25	10.12 *		5.72	3.95	6.73 *
26.38	9,12,15-Octadecatrienoic acid, ethyl ester, (Z,Z,Z)-		2.15		1.98				
26.46	9-Octadecenoic acid (Z)-, ethyl ester		9.96		3.38			2.26	
26.49	(E)-9-Octadecenoic acid ethyl ester						1.26		2.07
27.06	Octadecanoic acid, ethyl ester		6.31	1.58	4.01			2.37	1.85
34.09	Docosanoic acid, ethyl ester			1.28	1.47				
37.06	Ethyl tetracosanoate				1.02				
	**Other fatty acid esters**								
23.48	Isopropyl palmitate							1.14	
26.99	iso-Propyl 9-cis,11-trans-octadecadienoate							2.56	
30.95	9,12-Octadecadienoic acid (Z,Z)-, 2-hydroxy-1-(hydroxymethyl)ethyl ester	1.16							
35.13	(Z,Z)-9,12-octadeca-dienoic acid, 2,3-dihydroxy-propyl ester						3.89		
	**Fatty acid amides**								
29.68	9-Octadecenamide (Oleamide)	2.96						1.16	
36.42	13-Docosenamide, (Z)- (Erucamide)	1.76							
	**Sterols and phytosterols**								
42.64	(3-methyl,24R)-ergost-5-en-3-ol		1.25			2.84			2.06
42.67	Campesterol			8.03	5.19		3.40	4.98	
43.04	Stigmasta-5,22-dien-3-ol, (3 beta, 22E)-		2.82	8.78 *	8.41	8.78 *	6.41	6.52 *	4.92
43.75	.gamma.-Sitosterol			1.14				1.03	
	**Terpenoids/Isoprenoids**								
37.54	Squalene							1.09	
39.05	Oxirane, 2,2-dimethyl-3-(3,7,12,16,20-pentamethyl-3,7,11,15,19-heneicosapentaenyl)-, (all-E)-							1.92	
	**Vitamin E-related compounds (Tocols)**								
38.13	alpha-Tocospiro B			1.06					
41.41	dl-alpha-Tocopherol						1.17		

*: Major compound; DH-W-F-H: *D. hispida*-water washing-fresh-hexane; DH-W-F-E: *D. hispida*-water washing-fresh-ethanol; DH-W-D-H: *D. hispida*-water washing-dry-hexane; DH-W-D-E: *D. hispida*-water washing-dry-ethanol; DH-NW-F-H: *D. hispida*-non-water washing-fresh-hexane; DH-NW-F-E: *D. hispida*-non-water washing-fresh-ethanol; DH-NW-D-H: *D. hispida*-non-water washing-dry-hexane; DH-NW-D-E: *D. hispida*-non-water washing-dry-ethanol.

**Table 3:** Screening of antibacterial activity of *D. hispida* extracts using broth microdilution assay at a concentration of 2 mg/mL.

Extract and Control	Microorganisms		
	*S. aureus* ATCC 25923	*S. epidermidis* TISTR 517	*C. acnes* DMST 14916
1. DH-W-F-H	+	+	+
2. DH-W-F-E	+	+	+
3. DH-W-D-H	−	−	−
4. DH-W-D-E	+	+	+
5. DH-NW-F-H	−	−	−
6. DH-NW-F-E	−	−	−
7. DH-NW-D-H	−	−	−
8. DH-NW-D-E	−	−	−
Vancomycin (30 µg/mL)	+	+	+

+ indicates positive inhibition, whereas − indicates no inhibition.

**Table 4:** Minimal inhibitory concentration (MIC) and Minimal bactericidal concentration (MBC) of *D. hispida* extracts against three bacterial species.

Extract and Control	MIC/MBC Concentration (µg/mL)		
	*S. aureus* ATCC 25923	*S. epidermidis* TISTR 517	*C. acnes* DMST 14916
1. DH-W-F-H	64/>2048	2048/>2048	512/512
2. DH-W-F-E	1024/>2048	2048/>2048	1024/1024
3. DH-W-D-E	2048/>2048	2048/>2048	512/512
Vancomycin	0.125/0.50	0.125/0.50	0.25/0.25

**Table 5:** Comparison of binding energy and RMSD values of the tested compounds obtained from molecular docking and molecular dynamics simulations.

Compound	Binding Energy (kcal/mol)	Complex RMSD (nm)	Backbone RMSD (nm)
FA1	−55.66	0.256 ± 0.015	0.169 ± 0.015
FA2	−59.03	0.361 ± 0.051	0.253 ± 0.052
FA3	−57.66	0.282 ± 0.028	0.165 ± 0.022
Vancomycin	−58.39	0.325 ± 0.041	0.235 ± 0.037

**Table 6:** Energy components of the _CA_Lipase complex were calculated using the MM-GBSA approach from the last 50 ns of the trajectories. Data are shown as mean ± standard error mean (SEM).

Energies (kcal/mol)	_CA_Lipase-Van	_CA_Lipase-FA1	_CA_Lipase-FA2	_CA_Lipase-FA3
∆E_vdW_	−46.98 ± 0.26	−34.30 ± 0.14	−46.92 ± 0.16	−37.86 ± 0.14
∆E_ele_	−20.62 ± 0.43	−32.46 ± 0.33	−3.54 ± 0.07	−4.37 ± 0.14
∆G_gas_	−67.60 ± 0.57	−66.76 ± 0.31	−50.46 ± 0.20	−42.24 ± 0.23
${∆G}_{\mathrm{solv}}^{\mathrm{GB}}$	46.24 ± 0.44	36.89 ± 0.15	19.32 ± 0.08	16.02 ± 0.11
${∆G}_{\mathrm{solv}}^{\mathrm{nonpolar}}$	−5.36 ± 0.02	−5.46 ± 0.01	−6.90 ± 0.02	−5.71 ± 0.02
∆G_sol_	40.87 ± 0.43	31.43 ± 0.14	12.41 ± 0.06	10.30 ± 0.10
∆G_bind_	−26.73 ± 0.21	−35.33 ± 0.19	−38.05 ± 0.16	−31.93 ± 0.16

## Data Availability

The original contributions presented in this study are included in the article. Further inquiries can be directed to the corresponding author.

## Supplementary Materials

The following supporting information can be downloaded at: https://www.mdpi.com/article/10.3390/life16091494/s1, Figure S1: Broth microdilution assay for determination of the minimum inhibitory concentration (MIC) of *Dioscorea hispida* extracts against *Cutibacterium acnes*. Serial two-fold dilutions of the extracts (2048–4 µg/mL) were prepared in a 96-well microplate. Wells containing *C. acnes* without extract served as the bacterial growth control, while vancomycin was included as the positive control. The color change from blue or purple to light pink indicates bacterial growth. The MIC was defined as the lowest concentration of the extract that prevented detectable growth of *C. acnes*, as indicated by the absence of a color change to light pink; Figure S2: Representative gas chromatography–mass spectrometry (GC–MS) chromatogram of the *Dioscorea hispida* extract. The chromatogram shows the separation of volatile and semi-volatile constituents according to their retention times, with individual peaks representing detected compounds. The labeled peaks indicate the retention times of the major detected constituents.

## References

[1] Akaza N., Takasaki K., Nishiyama E., Usui A., Miura S., Yokoi A., Futamura K., Suzuki K., Yashiro Y., Yagami A. (2022). The Microbiome in Comedonal Contents of Inflammatory *Acne vulgaris* is Composed of an Overgrowth of *Cutibacterium* spp. and Other Cutaneous Microorganisms. Clin. Cosmet. Investig. Dermatol..

[2] Nonoguchi H.A., Liu G.Y., Hajam I.A. (2026). Microbial Hyaluronidases: From Obscure Virulence Factors to Promising Therapeutic Targets. Biomolecules.

[3] Boisrenoult P. (2018). *Cutibacterium acnes* Prosthetic Joint Infection: Diagnosis and Treatment. Orthop. Traumatol. Surg. Res..

[4] Wei M.P., Yu H., Guo Y.H., Cheng Y.L., Xie Y.F., Yao W.R. (2022). Synergistic Combination of Sapindoside A and B: A Novel Anti-Biofilm Agent Against *Cutibacterium acnes*. Microbiol. Res..

[5] Coenye T., Peeters E., Nelis H.J. (2007). Biofilm Formation by *Propionibacterium acnes* is Associated with Increased Resistance to Antimicrobial Agents and Increased Production of Putative Virulence Factors. Res. Microbiol..

[6] Gannesen A.V., Lesouhaitier O., Racine P.J., Barreau M., Netrusov A.I., Plakunov V.K., Feuilloley M.G.J. (2018). Regulation of Monospecies and Mixed Biofilms Formation of Skin *Staphylococcus aureus* and *Cutibacterium acnes* by Human Natriuretic Peptides. Front. Microbiol..

[7] Jahns A.C., Lundskog B., Ganceviciene R., Palmer R.H., Golovleva I., Zouboulis C.C., McDowell A., Patrick S., Alexeyev O.A. (2012). An Increased Incidence of *Propionibacterium acnes* Biofilms in *Acne vulgaris*: A case-control study. Br. J. Dermatol..

[8] Kuehnast T., Cakar F., Weinhäupl T., Pilz A., Selak S., Schmidt M.A., Rüter C., Schild S. (2018). Comparative Analyses of Biofilm Formation Among Different *Cutibacterium acnes* Isolates. Int. J. Med. Microbiol..

[9] Bjerg C.S.B., Poehlein A., Bömeke M., Himmelbach A., Schramm A., Brüggemann H. (2024). Increased Biofilm Formation in Dual-Strain Compared to Single-Strain Communities of *Cutibacterium acnes*. Sci. Rep..

[10] Rieger U.M., Mesina J., Kalbermatten D.F., Haug M., Frey H.P., Pico R., Frei R., Pierer G., Lüscher N.J., Trampuz A. (2013). Bacterial Biofilms and Capsular Contracture in Patients with Breast Implants: Breast Capsular Contracture and Bacterial Biofilm. Br. J. Surg..

[11] Farhat-Sabet A., Hull R., Thomas D. (2018). Cardiac Tamponade from Purulent Pericarditis Due to *Cutibacterium acnes*. Case Rep. Cardiol..

[12] Dutta B. (2015). Food and Medicinal Values of Certain Species of *Dioscorea* with Special Reference to Assam. J. Pharmacogn. Phytochem..

[13] Estiasih T., Ahmadi K., Sari I.N.I., Kuliahsari D.E., Martati E. (2022). Traditional Detoxification of Wild Yam (*Dioscorea hispida* Dennst.) Tuber in Chips Processing at East Java, Indonesia. J. Ethn. Foods.

[14] Wang P., Shan N., Ali A., Sun J., Luo S., Xiao Y., Zhang H., Li X., Chen Y., Liu J. (2022). Comprehensive Evaluation of Functional Components, Biological Activities, and Minerals of Yam Species (*Dioscorea polystachya* and *D. alata*) from China. LWT.

[15] Syahputra H., Fadila N., Siringoringo Y.L. (2025). Phytochemical Screening, Antioxidant, and Anti-Inflammatory Activities of *Dioscorea hispida* (Dennst.) Tuber Extracts and Fractions. Res. J. Pharm. Technol..

[16] Avula B., Wang Y.H., Wang M., Ali Z., Smillie T.J., Zweigenbaum J., Khan I.A. (2014). Characterization of Steroidal Saponins from *Dioscorea villosa* and *D. Cayenensis* Using Ultrahigh Performance Liquid Chromatography/Electrospray Ionization Quadrupole Time-Offlight Mass Spectrometry. Planta Medica.

[17] Price E.J., Wilkin P., Sarasan V., Fraser P.D. (2016). Metabolite Profiling of *Dioscorea* (yam) Species Reveals Underutilised Biodiversity and Renewable Sources for High-Value Compounds. Sci. Rep..

[18] Lal S., Mahendru N., Malhotra C., Sable P.D., Ofoeze M.A., Kumar S. (2024). Pharmaceutical and Nutraceutical Values of *Dioscorea hispida* Dennst.: A Wild Food of Asia and Africa. Acta Fytotech. Zootech..

[19] Casillas-Vargas G., Ocasio-Malavé C., Medina S., Morales-Guzmán C., Del Valle R.G., Carballeira N.M., Sanabria-Ríos D.J. (2021). Antibacterial Fatty Acids: An Update of Possible Mechanisms of Action and Implications in the Development of the Next-Generation of Antibacterial Agents. Prog. Lipid Res..

[20] Paul C., Chakraborty K., Bhattacharjee A., Debnath B. (2022). Gender-Specific Differences in Micro-Morphology, Secondary Metabolites, Antioxidant and Antimicrobial Activity of Wild *Dioscorea* species. Vegetos.

[21] Ghosh S., Nitnavare R., Dewle A., Tomar G.B., Chippalkatti R., More P., Kitture R., Kale S., Gohil S., Bellare J. (2015). Novel Platinum–Palladium Bimetallic Nanoparticles Synthesized by *Dioscorea bulbifera*: Anticancer and Antioxidant Activities. Int. J. Nanomed..

[22] Sangkanu S., Khanansuk J., Phoopha S., Udomuksorn W., Phupan T., Puntarat J., Tungsukruthai S., Dej-adisai S. (2025). Utility Assessment of Isolated Starch and Extract from Thai Yam (*Dioscorea hispida* Dennst.) for Cosmetic via In Vitro and In Vivo Studies. Life.

[23] (2012). Methods for Dilution Antimicrobial Susceptibility Tests for Bacteria that Grow Aerobically.

[24] Sarker S.D., Nahar L., Kumarasamy Y. (2007). Microtitre Plate-Based Antibacterial Assay Incorporation Resazurin as An Indicator of Cell Growth, and Its Application in the In Vitro Antibacterial Screening of Phytochemicals. Methods.

[25] Ruchiatan K., Rizqandaru T., Satjamanggala P.R., Tache N., Cahyadi A.I., Rezano A., Gunawan H., Sutedja E.K., Dwiyana R.F., Hidayah R.M.N. (2023). Characteristics of Biofilm-Forming Ability and Antibiotic Resistance of *Cutibacterium acnes* and *Staphylococcus epidermidis* from Acne Vulgaris Patients. Clin. Cosmet. Investig. Dermatol..

[26] Christensen A.S., Kubar T., Cui Q., Elstner M. (2016). Semiempirical Quantum Mechanical Methods for Noncovalent Interactions for Chemical and Biochemical Applications. Chem. Rev..

[27] Wang J., Wolf R.M., Caldwell J.W., Kollman P.A., Case D.A. (2004). Development and Testing of a General AMBER Force Field. J. Comput. Chem..

[28] Maier J.A., Martinez C., Kasavajhala K., Wickstrom L., Hauser K.E., Simmerling C. (2015). ff14SB: Improving the Accuracy of Protein Side Chain and Backbone Parameters from ff99SB. J. Chem. Theory Comput..

[29] Brozell S.R., Mukherjee S., Balius T.E., Roe D.R., Case D.A., Rizzo R.C. (2012). Evaluation of DOCK 6 as A Pose Generation and Database Enrichment Tool. J. Comput. Aided Mol. Des..

[30] Allen W.J., Balius T.E., Mukherjee S., Brozell S.R., Moustakas D.T., Lang P.T., May A., Ten Eyck L.F., Kuntz I.D., Rizzo R.C. (2015). DOCK 6: Impact of New Features and Current Docking Performance. J. Comput. Chem..

[31] Case D.A., Aktulga H.M., Belfon K., Cerutti D.S., Cisneros G.A., Cruzeiro V.W.D., Brozell S.R., Gohlke H., Goetz A.W., Harris R. (2023). AmberTools. J. Chem. Inf. Model..

[32] Case D.A., Cheatham T.E., Darden T., Gohlke H., Luo R., Merz K.M., Onufriev A., Simmerling C., Wang B., Woods R.J. (2005). The AMBER Biomolecular Simulation Programs. J. Comput. Chem..

[33] Lee T.S., Cerutti D.S., Mermelstein D., Lin C., LeGrand S., Giese T.J., Roitberg A., Case D.A., Walker R.C., York D.M. (2018). GPU-Accelerated Molecular Dynamics and Free Energy Methods in AMBER18: Performance Enhancements and New Features. J. Chem. Inf. Model..

[34] Roe D.R., Cheatham T.E. (2013). PTRAJ and CPPTRAJ: Software for Processing and Analysis of Molecular Dynamics Trajectory Data. J. Chem. Theory Comput..

[35] Grant B.J., Rodrigues A.P.C., ElSawy K.M., McCammon J.A., Caves L.S. (2006). Bio3D: An R Package for the Comparative Analysis of Protein Structures. Bioinformatics.

[36] Miller B.R., McGee T.D., Swails J.M., Homeyer N., Gohlke H., Roitberg A.E. (2012). MMPBSA.py: An Efficient Program for End-State Free Energy Calculations. J. Chem. Theory Comput..

[37] Chandrasekaran M., Kannathasan K., Venkatesalu V. (2008). Antimicrobial Activity of Fatty Acid Methyl Esters of Some Members of Chenopodiaceae. Z. Für Naturforschung. C J. Biosci..

[38] Câmara J.S., Perestrelo R., Ferreira R., Berenguer C.V., Pereira J.A.M., Castilho P.C. (2024). Plant-Derived Terpenoids: A Plethora of Bioactive Compounds with Several Health Functions and Industrial Applications—A Comprehensive Overview. Molecules.

[39] Kozłowska M., Ścibisz I., Przybył J., Ziarno M., Żbikowska A., Majewska E. (2021). Phenolic Contents and Antioxidant Activity of Extracts of Selected Fresh and Dried Herbal Materials. Pol. J. Food Nutr. Sci..

[40] Mustafa I., Chin N.L. (2023). Antioxidant Properties of Dried Ginger (*Zingiber officinale* Roscoe) var. *Bentong*. Foods.

[41] Kim H.K., Verpoorte R. (2010). Sample Preparation for Plant Metabolomics. Phytochem. Anal..

[42] Krakowska-Sieprawska A., Kiełbasa A., Rafińska K., Ligor M., Buszewski B. (2022). Modern Methods of Pre-Treatment of Plant Material for the Extraction of Bioactive Compounds. Molecules.

[43] Saini R.K., Prasad P., Shang X., Keum Y.S. (2021). Advances in Lipid Extraction Methods—A review. Int. J. Mol. Sci..

[44] Yoon B.H., Truong V.L., Jeong W.S. (2025). Phytosterols: Extraction Methods, Analytical Techniques, and Aiological activity. Molecules.

[45] Alves E., Dias M., Lopes D., Almeida A., Domingues M.D.R., Rey F. (2020). Antimicrobial Lipids from Plants and Marine Organisms: An overview of the Current State-Of-The-Art and Future Prospects. Antibiotics.

[46] Shen M., Yuan L., Zhang J., Wang X., Zhang M., Li H., Jing Y., Zeng F., Xie J. (2024). Phytosterols: Physiological Functions and Potential Application. Foods.

[47] Rutherford S.T., Bassler B.L. (2012). Bacterial Quorum Sensing: Its Role in Virulence and Possibilities for Its Control. Cold Spring Harb. Perspect. Med..

[48] Kim Y.G., Lee J.H., Lee J. (2021). Anti-Biofilm Activities of Fatty Acids Including Myristoleic Acid against *Cutibacterium acnes* via Reduced Cell Hydrophobicity. Phytomedicine.

[49] Lin M., He L., Ye G., Zhao X. (2025). Computational Methodology and Molecular Dynamics Analysis of Andrographolide Bioactivity against *Cutibacterium acnes*. Front. Chem..

[50] Hollingsworth S.A., Dror R.O. (2018). Molecular Dynamics Simulation for All. Neuron.

[51] Wang E., Sun H., Wang J., Wang Z., Liu H., Zhang J.Z., Hou T. (2019). End-Point Binding Free Energy Calculation with MM/PBSA and MM/GBSA: Strategies and Applications in Drug Design. Chem. Rev..

[52] LaPlante K.L., Mermel L.A. (2009). In vitro Activities of Telavancin and Vancomycin against Biofilm-Producing *Staphylococcus aureus*, *S. epidermidis*, and *Enterococcus faecalis* Strains. Antimicrob. Agents Chemother..

[53] El-Azizi M., Rao S., Kanchanapoom T., Khardori N. (2005). In vitro Activity of Vancomycin, Quinupristin/Dalfopristin, and Linezolid against Intact and Disrupted Biofilms of staphylococci. Ann. Clin. Microbiol. Antimicrob..

