# Supplementary material for: From Detoxified Yam to Bioactive Extracts: Integrated Extraction and In Silico Evidence of Anti-Biofilm Activity of Dioscorea hispida Extracts Against Cutibacterium acnes
Source: Life (Basel). 2026 Sep 6;16(9):1494. doi: 10.3390/life16091494 (PMC13608383; doi:10.3390/life16091494)
Supplement: Supplementary file 1 [file life-16-01494-s001.zip › life-4511730-supplementary.pdf]

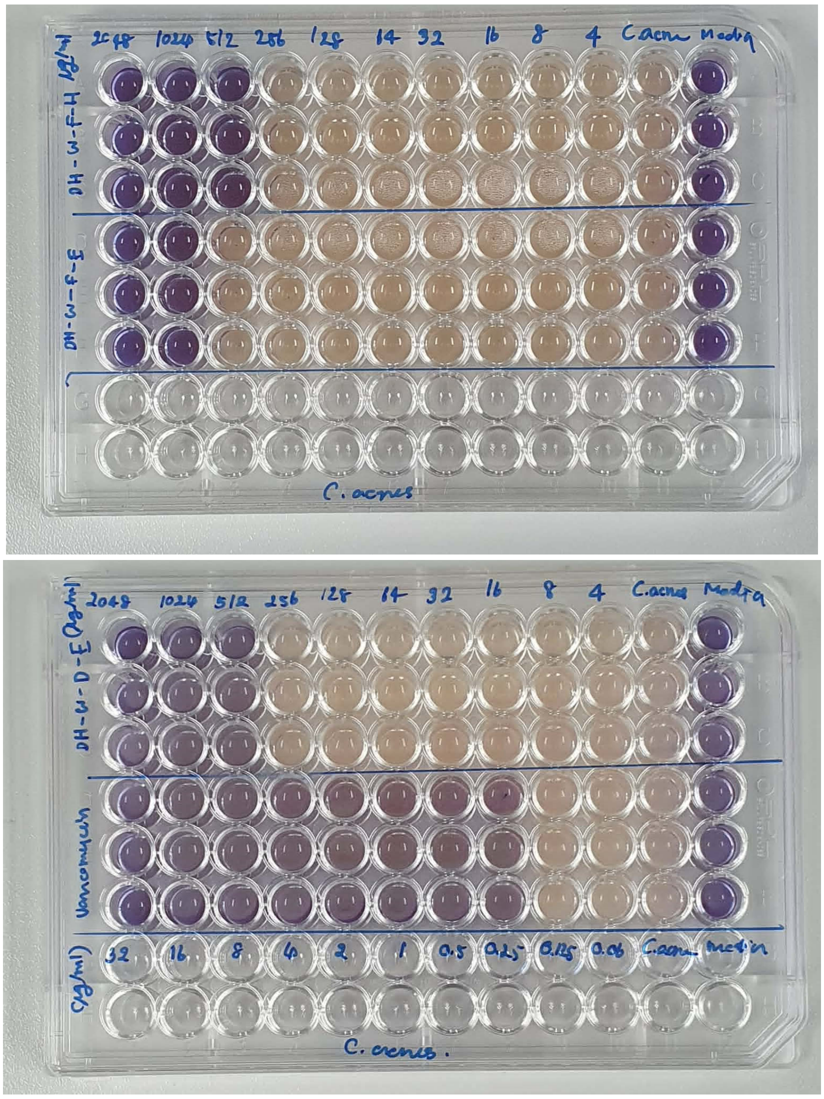

Figure S1 Broth microdilution assay for determination of the minimum inhibitory concentration (MIC) of *Dioscorea hispida* extracts against *Cutibacterium acnes*. Serial two-fold dilutions of the extracts (2,048–4 µg/mL) were prepared in a 96-well microplate. Wells containing *C. acnes* without extract served as the bacterial growth control, while vancomycin was included as the positive control. The color change from blue or purple to light pink indicates bacterial growth. The MIC was defined as the lowest concentration of the extract that prevented detectable growth of *C. acnes*, as indicated by the absence of a color change to light pink.

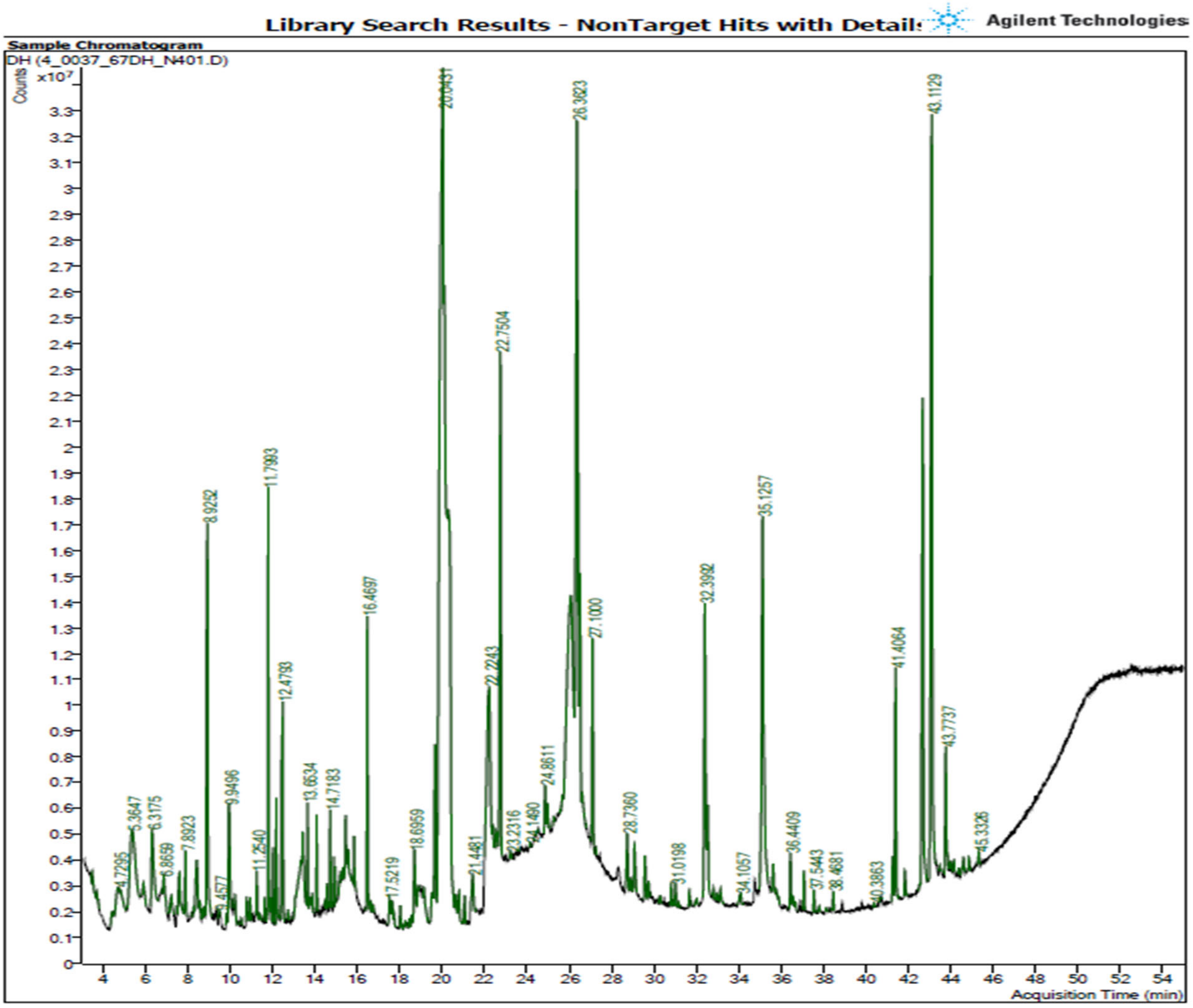

Figure S2. Representative gas chromatography–mass spectrometry (GC–MS) chromatogram of the *Dioscorea hispida* extract. The chromatogram shows the separation of volatile and semi-volatile constituents according to their retention times, with individual peaks representing detected compounds. The labeled peaks indicate the retention times of the major detected constituents.
